# LogoRef: A Region-Aware Framework for Reference-Based Logo Sketch Colorization

**DOI:** 10.3390/s26196140

**Published:** 2026-09-28

**Authors:** Nannan Tian, Zhuolong Jiang

**Affiliations:** 1 School of Design and Art, Shanghai DianJi University, Shanghai 201306, China; 2 School of Computer Science, Shanghai Jiao Tong University, Shanghai 200240, China; jzz19961996@sjtu.edu.cn

**Keywords:** logo sketch colorization, reference-based colorization, region-aware colorization, style transfer

## Abstract

Reference-based logo colorization aims to generate controllable color schemes while preserving clear contours and consistent colors within graphic regions. However, existing colorization and style transfer methods often suffer from ambiguous color assignment, inconsistent filling, and cross-boundary color interference when applied to logos with compact structures and nested regions. To address these issues, we propose LogoRef, a reference-based logo colorization framework that integrates sketch structure preservation, reference color extraction, and region-aware color assignment. First, we propose a Hierarchical Region Prior (HRP) to extract region structures at different levels from the input line art. Second, we introduce the Semantic Region Style Attention module, which constructs a reference style library and adaptively selects appropriate color statistics for different logo regions, thereby improving intra-region color consistency and reducing color interference between adjacent regions. In addition, we construct JN-Logo-v2, a high-quality logo image dataset that provides richer and more reliable data support for logo colorization. Finally, we propose two task-specific evaluation metrics, Color Fidelity and Boundary Integrity, to evaluate reference color consistency and boundary-aware colorization quality, respectively. Extensive experiments on JN-Logo-v2 and a public anime sketch colorization dataset demonstrate that our method achieves superior performance in both quantitative and qualitative comparisons.

## 1. Introduction

Traditional logo design requires designers to collect materials, draw graphics and text, select color schemes, and manually fill different regions. This process is time-consuming and requires professional experience. With growing online and brand design demands, automatic logo colorization has become important for improving design efficiency. Color affects visual attractiveness and brand recognition. Unlike natural images, logos usually contain compact graphics, clear contours, and large uniform color regions. Logo colorization should therefore generate appropriate colors while preserving region boundaries, maintaining intra-region consistency, and following the reference image.

Existing deep learning methods have achieved considerable progress, but still show limitations for logos. Automatic colorization and image translation methods learn mappings from grayscale or structural inputs to color images [1,2,3]. However, they usually do not use reference images and thus provide limited control, often producing ambiguous or inconsistent colors. Style transfer methods obtain color and appearance information from references [4,5,6], but mainly focus on textures and global visual characteristics. Direct application may introduce unnecessary textures and local color variations. Reference-based line drawing colorization methods transfer colors by learning correspondences between sketches and references [7,8,9]. Most are designed for anime illustrations with shading and gradients, which differ from the flat regions and clean boundaries required by logos. Region-guided methods use region maps, spatial masks, or skeleton maps to reduce color bleeding [10,11,12], but often rely on annotated regions, task-specific extraction, or region representations with limited semantic information. Accurately selecting reference colors for logo regions while maintaining uniform filling and clear boundaries therefore remains challenging.

To address these issues, we propose LogoRef, a reference-based framework for logo colorization. Given a sketch and a reference image, we first construct a hierarchical region prior (HRP) map directly from the sketch to provide explicit structural guidance for different logo regions. A Color Style Extractor then obtains global color information and spatial style features from the reference image. The generator takes the sketch as input and uses skip connections to preserve thin lines and text contours. During decoding, global color information controls the overall color scheme, while the proposed Semantic Region Style Attention (SRSA) combines reference style features with the HRPs to select suitable reference color statistics for different logo regions. Multi-scale outputs are trained with reconstruction and adversarial losses.

The HRP is derived directly from the sketch and represents region relationships at different levels, providing explicit structural guidance for color assignment. Based on the HRP, SRSA constructs a reference style library and selects suitable color statistics for different regions, thereby improving intra-region color consistency and reducing color interference across boundaries. To support training and evaluation, we construct JN-Logo-v2 based on JN-Logo [13], retaining 6000 high-quality logo images after data screening. We further introduce two task-specific metrics, Color Fidelity (CF) and Boundary Integrity (BI), to evaluate reference color consistency and the alignment between color variations and sketch boundaries, respectively.

To validate the proposed method, we conduct extensive experiments on JN-Logo-v2 and a public anime sketch colorization dataset. Quantitative and qualitative comparisons demonstrate that our method achieves superior colorization performance while better preserving structural boundaries and reference color consistency. Ablation studies and user evaluations further verify the effectiveness of the HRP and SRSA.

Our main contributions are summarized as follows:

- We construct JN-Logo-v2, a refined logo colorization dataset, providing a reliable data foundation for training and evaluating logo colorization models.
- We introduce two task-specific metrics, CF and BI, to evaluate reference color consistency and the alignment between color variations and sketch boundaries, respectively.
- We propose LogoRef, a reference-based logo colorization framework that integrates reference color extraction, sketch structure preservation, region-aware color assignment, and multi-scale training for controllable logo colorization. In addition, an HRP is derived directly from logo sketches to explicitly encode region structures at different levels and provide stable structural guidance without relying on an external segmentation model.
- We propose SRSA, which models relationships among different logo regions and selects suitable color statistics from the reference style library for each region, enabling more accurate region-level color assignment while improving intra-region color consistency and reducing color interference between adjacent regions.

## 2. Background and Related Work

Image colorization aims to generate plausible colors while preserving the structure of an input image. For logo sketches, the task further requires clear contours, uniform colors within graphic regions, and controllable color schemes. Existing studies related to this work can be grouped into image colorization, style transfer, line drawing colorization, and region-based colorization.

### 2.1. Image Colorization

Early deep learning methods learn a direct mapping from grayscale or structural inputs to color images. The conditional image translation framework pix2pix [1] employs a U-Net generator and a PatchGAN discriminator to learn paired mappings, whereas CycleGAN [2] introduces cycle consistency for unpaired domain translation. These frameworks are widely used as general baselines because they can learn the overall appearance of a target domain without manually designed color propagation rules. To improve semantic and object-level consistency, Su et al. [14] introduce instance-aware colorization, in which object-level features extracted from detected instances are fused with full-image features. Palette-based methods provide another form of color control. PalGAN [15] models colorization through a palette generative adversarial framework, while BigColor [16] exploits a generative color prior to produce vivid and semantically plausible colors for natural images. More recently, DDColor [3] adopts a pixel decoder and a query-based color decoder to associate semantic representations with colors and alleviate color bleeding. Although these methods improve semantic plausibility and color diversity, they mainly infer colors from the input itself or from a global color prior. They do not explicitly establish region-level correspondence with a given reference image, which can lead to ambiguous colors or inconsistent filling when applied to logos containing multiple adjacent regions.

### 2.2. Style Transfer

Style transfer methods synthesize an output by preserving the structure of a content image while transferring visual characteristics from a reference image. Feature statistics-based methods provide an important foundation for efficient arbitrary style transfer. Adaptive instance normalization (AdaIN) [17] aligns the channel-wise mean and variance of content features with those of style features, while the whitening and coloring transform (WCT) [18] matches higher-order feature statistics through whitening and covariance-based coloring transformations. Later methods improve the representation and interaction of content and style information. StyleFormer [4] performs arbitrary style transfer through parametric style composition, improving both fine-grained style diversity and content coherence. ${\mathrm{StyTr}}^{2}$ [5] uses separate Transformer encoders for content and style features and a Transformer decoder to model their long-range interactions. TSSAT [6] first aligns global feature statistics and then exchanges local statistics to preserve the overall style while enriching local patterns. These methods demonstrate that reference statistics and feature interactions can effectively control visual appearance. However, general style transfer mainly focuses on textures, brush patterns, and global or patch-level statistics, whereas logo colorization requires flat and uniform colors, accurate correspondence between graphic regions, and strict preservation of clean contours. Directly applying global or texture-oriented style transformation may therefore introduce unwanted color variations or assign reference colors to incorrect logo regions.

### 2.3. Line Drawing Colorization

Line drawings contain sparse structural information but little luminance or texture information, making their colorization more difficult than grayscale image colorization. User-guided methods use scribbles, tags, or palettes as color conditions [19,20], while reference-based methods transfer colors from a given image by learning semantic correspondences [7,8]. For icon images, Sun et al. [21] jointly use contour and color conditions in an adversarial framework, and Li et al. [22] disentangle style and structure with normalizing flows to generate diverse icon colorizations. The attention-aware method proposed by Cao et al. [23] combines channel and spatial attention with cross-domain attention to improve color consistency and semantic correspondence. AnimeDiffusion [9] further introduces a diffusion model with a two-phase training strategy for reference-based anime face line drawing colorization, while FlexIcon [24] uses guided images and palettes to provide flexible color control for icons. These studies demonstrate the effectiveness of structural guidance, reference information, and learned correspondence. Nevertheless, anime- and illustration-oriented approaches often model shading, gradients, textures, or character-level appearance, whereas logo images mainly consist of compact shapes and large uniform color regions. For logo colorization, reference colors must be assigned consistently to structurally distinct regions without introducing unnecessary texture or cross-boundary color leakage.

### 2.4. Region-Based Colorization

Region information provides an effective constraint for preventing colors from crossing structural boundaries. DanbooRegion [25] provides paired illustrations and region maps for learning line drawing regions, while the split filling mechanism [26] estimates the influence areas of grouped color hints to reduce cross-region bleeding. For temporally consistent line-art colorization, Transformation Region Enhancement Network (TRE-Net) [27] localizes geometric transformation regions through multi-scale edge-based maps and enhances these regions to improve color alignment. Region-Assisted Sketch Colorization (RASC) [10] further treats region maps as intermediate representations and models local styles and inter-region dependencies in a region-wise manner. Yan et al. [11] use spatial masks and split cross-attention to reduce mismatched reference information, and He et al. [12] introduce skeleton maps as additional guidance for diffusion-based line drawing colorization. These methods show that explicit region information improves boundary preservation, correspondence, and local color consistency. However, many existing approaches rely on manually annotated masks, task-specific region extraction, or externally provided region maps. For logo sketches, how to derive stable HRPs directly from sparse line structures and use them to guide the selection of reference color statistics remains insufficiently explored.

## 3. Method

### 3.1. Overview

As shown in Figure 1, the proposed framework takes a logo sketch $I_{sk}$ and a reference color image $I_{ref}$ as inputs. An HRP map $I_{reg}$ is first constructed directly from the sketch to describe the structural relationships among different logo regions. The Color Style Extractor obtains a style code *s* and a spatial style feature *S* from $I_{ref}$. The sketch is then processed by a generator with skip connections. During decoding, AdaIN provides the overall color style, while the proposed SRSA modules use *S* and the HRPs to select suitable reference colors for different logo regions. The generator produces colorized images at multiple resolutions, which are evaluated by a multi-scale discriminator during training.

### 3.2. Hierarchical Region Prior

Logo sketches usually consist of compact graphic regions enclosed by clear contours, and these regions often exhibit nested structures. To provide explicit structural guidance without relying on an external segmentation model, we construct a hierarchical region prior (HRP) directly from $I_{sk}$. The prior describes both the separation of individual regions and their outer-to-inner relationships, providing stable structural information for logo colorization.

As shown in Figure 2, the construction process mainly consists of three stages: sketch processing, region extraction, and hierarchical region encoding. Starting from the input sketch, the contour structure is first enhanced to obtain reliable closed boundaries. Individual regions are then extracted according to these boundaries. Finally, the extracted regions are organized according to their outer-to-inner relationships and encoded into the HRP.

Specifically, given the sketch $I_{sk}$, we first extract the dark sketch lines to obtain a binary line mask *L*:

(1) $$L\left(i,j\right)=I\left(I_{sk}\left(i,j\right)\leq \tau \right),$$

where I(·) denotes the indicator function and τ is the threshold for extracting sketch lines. In our implementation, τ is determined by Otsu’s method for each sketch, followed by one 3×3 morphological closing operation to remove small contour gaps.

Based on the processed sketch contours, connected components are extracted from the non-line area. All components touching the image border are merged into the external background region $\Omega_{0}$, while the remaining components are treated as individual logo regions ${\left\{\Omega_{k}\right\}}_{k=1}^{M}$. Regions smaller than 8 pixels are merged into the non-background neighboring region with the longest shared boundary.

After obtaining the individual regions, we further determine their hierarchical relationships. An undirected adjacency graph G=(V,E) is constructed, where each vertex $v_{k}\in V$ corresponds to a region $\Omega_{k}$, and an edge is created when two regions are spatially adjacent across a sketch contour. Taking the external background $v_{0}$ as the root, the hierarchical level of region $\Omega_{k}$ is defined by its shortest graph distance from the background:

(2) $$d_{k}=\min_{\pi :v_{0}⇝v_{k}}\left|\pi \right|,\;d_{0}=0,$$

where |π| denotes the number of region transitions along path π. Regions closer to the external background therefore receive smaller hierarchical levels, while regions enclosed by other regions obtain progressively larger levels.

Multiple disconnected regions at the same hierarchical level remain independent and are deterministically ordered according to their spatial position and area. They share the same hierarchical level while being assigned slightly different structural codes, allowing regions with the same nesting depth to remain distinguishable. The resulting HRP is expressed as

(3) $$I_{\mathrm{reg}}\left(i,j\right)=C\left(d_{k},r_{k}\right),\;\left(i,j\right)\in \Omega_{k},$$

where $r_{k}$ denotes the within-level region index, and C(·) maps the hierarchical level and region index to a stable structural code. These codes represent only region structures and do not correspond to the target logo colors.

Finally, the HRP is converted into at most *K* region masks for network processing. Each structural code is converted into a binary mask ${\tilde{R}}_{k}$. If more than *K* regions are obtained, the *K* largest regions are retained; otherwise, zero masks are added. The masks are normalized as

(4) $$R_{k}\left(i,j\right)=\frac{{\tilde{R}}_{k}\left(i,j\right)}{\sum_{m=1}^{K}{\tilde{R}}_{m}\left(i,j\right)+\epsilon },\;k=1,\ldots ,K,$$

where ϵ is a small constant used to avoid numerical instability. The resulting HRPs are denoted as $R\in R^{K\times H\times W}$ and are resized to the required spatial resolution during network processing.

Because HRP applies one 3×3 morphological closing operation before region extraction, small local gaps in binary contours can often be connected and therefore have limited influence on the extracted regions. For more pronounced contour breaks, however, the connectivity of local regions may change and affect the resulting HRP partition. The subsequent SRSA module further combines region features and predicts a confidence map at uncertain boundaries, which reduces the influence of unreliable region information during color assignment. Nevertheless, severe contour discontinuities that substantially alter the original region topology remain a failure case for contour-derived HRPs.

### 3.3. Reference-Based Colorization Network

#### 3.3.1. Color Style Extractor

The Color Style Extractor consists of an initial convolutional layer followed by four residual downsampling blocks. Given $I_{ref}$, the last block produces a spatial style feature *S*, which preserves the color information at different reference locations. Global average pooling (GAP) and a fully connected (FC) layer are then used to obtain a 256-dimensional style code:

(5) $$s=\mathrm{FC}\left(\mathrm{GAP}(S)\right).$$

The style code *s* provides the overall color style for the decoder, while *S* is used by SRSA to construct the reference style library.

#### 3.3.2. Colorization Generator

The colorization generator adopts a symmetric U-Net with five downsampling blocks and five upsampling blocks. The encoder takes only $I_{sk}$ as input and progressively extracts its structural features through convolution, instance normalization, and rectified linear unit (ReLU) activation. The decoder restores the spatial resolution through upsampling and convolution. Skip connections transfer encoder features to the corresponding decoder stages, helping preserve thin lines, text contours, and small graphic details.

Each decoder block uses *s* through AdaIN. In selected stages, the second AdaIN operation is replaced by SRSA, which additionally receives *S* and *R*. Four output layers generate colorized results at resolutions of 32×32, 64×64, 128×128, and 256×256. The full-resolution result is used as the final output, while all four outputs participate in training.

### 3.4. Semantic Region Style Attention

A single global style representation applies the same color statistics to all spatial locations. This is insufficient for logo images, since different graphic regions may require different colors while their boundaries should remain clear. To improve the correspondence between the reference image and individual logo regions, we propose the Semantic Region Style Attention (SRSA) module, as shown in Figure 1. SRSA first extracts several sets of color statistics from the reference feature and then selects suitable statistics for each region according to the decoder feature and the HRPs.

Let $F\in R^{B\times C\times H\times W}$ be the decoder feature, *S* the reference style feature, $s\in R^{B\times d}$ the style code, and $R\in R^{B\times K\times H\times W}$ the normalized HRPs. We retain an AdaIN branch to obtain a stable base feature. The decoder feature is first normalized as

(6) $$\hat{F}=\frac{F-\mu (F)}{\sigma (F)+\epsilon }.$$

The style code is passed through a multilayer perceptron (MLP) to predict the scale $\gamma_{s}$ and bias $\beta_{s}$, and the base feature is calculated by

(7) $$F_{\mathrm{base}}=\hat{F}⊙\left(1+\gamma_{s}\right)+\beta_{s}.$$

To construct the reference style library, *S* is projected to the same channel dimension as *F*. A learnable assignment layer followed by Softmax divides the reference feature into *P* weighted groups. For the *p*th group, its mean and standard deviation are computed as

(8) $$\begin{array}{cc} \mu_{p} & =\sum_{n}A_{p,n}{\bar{S}}_{n},\\ \sigma_{p} & =\sqrt{\sum_{n}A_{p,n}{\bar{S}}_{n}^{2}-\mu_{p}^{2}+\epsilon }. \end{array}$$

where $\bar{S}$ is the projected reference feature and *A* denotes the normalized assignment weights. The resulting library $L={\left\{\left(\mu_{p},\sigma_{p}\right)\right\}}_{p=1}^{P}$ preserves several color distributions from the reference image instead of using only one global mean and standard deviation.

Next, the HRPs are used to perform average pooling on the decoder feature. The feature of the *k*th region is given by

(9) $$f_{k}=\frac{\sum_{i,j}R_{k}\left(i,j\right)F\left(:,i,j\right)}{\sum_{i,j}R_{k}\left(i,j\right)+\epsilon },\;k=1,\ldots ,K.$$

All region features are projected by an MLP and processed by multi-head self-attention. In this way, the color selection of one region can consider the other regions in the same logo. For each updated region feature ${\tilde{f}}_{k}$, an MLP predicts its selection weights over the reference style library:

(10) $$w_{k}=\mathrm{Softmax}\left(\mathrm{MLP}(\left[{\tilde{f}}_{k};s\right])\right).$$

The mean and standard deviation selected for this region are

(11) $$\left(\mu_{k},\sigma_{k}\right)=\sum_{p=1}^{P}w_{k,p}\left(\mu_{p},\sigma_{p}\right).$$

The selected statistics are restored to their corresponding spatial regions to form $\mu_{R}$ and $\sigma_{R}$, and the region-aware feature is obtained by

(12) $$F_{\mathrm{reg}}=\hat{F}⊙\sigma_{R}+\mu_{R}.$$

Although the HRP is constructed from explicit sketch contours, small gaps or local fragments may still introduce uncertainty around some region boundaries. SRSA therefore predicts a confidence map *G* from *F* and *R*. The region-aware result is then added to the base feature in a residual form:

(13) $$F_{\mathrm{sem}}=\alpha G⊙\left(F_{\mathrm{reg}}+F_{\mathrm{base}}\right),$$

(14) $$F_{\mathrm{out}}=F_{\mathrm{base}}+F_{\mathrm{sem}}.$$

where α is a learnable coefficient initialized to zero. Therefore, SRSA preserves the stable global color information provided by AdaIN while introducing region-specific reference colors at reliable locations. This improves color consistency within each logo region and reduces color bleeding across region boundaries.

### 3.5. Loss Functions

The generator produces four outputs ${\left\{{\hat{y}}_{i}\right\}}_{i=1}^{N}$, where N=4, and the ground-truth logo is resized to the corresponding resolution as $y_{i}$. We use a multi-scale reconstruction loss containing a VGG perceptual loss and a mean squared error (MSE) loss:

(15) $$L_{\mathrm{rec}}=\frac{1}{N}\sum_{i=1}^{N}\lambda_{i}\left(L_{\mathrm{perc}}^{i}+\omega_{1}L_{\mathrm{MSE}}^{i}\right).$$

Here, $L_{\mathrm{perc}}^{i}$ measures the squared distance between VGG features, and $L_{\mathrm{MSE}}^{i}$ constrains the pixel values. The scale weights are set to {1,2,3,10}.

The same discriminator is applied to all four resolutions. The generator adversarial loss and the total generator loss are

(16) $$L_{\mathrm{adv}}=-\frac{1}{N}\sum_{i=1}^{N}\lambda_{i}D\left({\hat{y}}_{i}\right).$$

(17) $$L_{G}=L_{\mathrm{rec}}+L_{\mathrm{adv}}.$$

The discriminator is trained with hinge loss on real and generated images, together with a gradient penalty on real images. These loss functions encourage accurate colors, clear contours, and visually realistic colorized logos.

Finally, the complete training objective consists of the generator and discriminator losses:

(18) $$L_{\mathrm{total}}=L_{G}+L_{D},$$

where $L_{D}$ denotes the discriminator loss. The generator and discriminator are optimized alternately during training.

## 4. Evaluation

Additional analyses of the SRSA reference style library size, cross-image reference colorization, controlled validation of CF and BI, HRP sensitivity and robustness, and metric sensitivity, robustness, and correlation with human judgments are provided in Sections S1–S5 of the Supplementary Materials.

### 4.1. Experimental Setup

#### 4.1.1. Datasets

We conduct experiments on two datasets, including our JN-Logo-v2 dataset and the publicly available Anime Sketch Colorization Pair Dataset.

**JN-Logo-v2:** We construct JN-Logo-v2 based on the JN-Logo dataset [13]. First, following consistent data-quality criteria, we manually screen more than 14,000 images in the original dataset and remove low-resolution, duplicate, visually poor, overly simple, or otherwise unsuitable samples for sketch colorization, ultimately retaining 6000 high-quality Logo images. We then manually select 300 representative images covering different levels of structural complexity, numbers of regions, and color styles as the test set, while the remaining 5700 images are used for training. The test images are selected solely according to predefined sample-quality and diversity criteria, without considering model performance or experimental results, and remain unseen during model training and parameter tuning. Although the 300-image test set may limit coverage of extremely rare structural or color patterns, its broad representativeness, together with the use of the same fixed data split for all compared methods, makes its influence on the overall performance trends and main conclusions relatively limited. Each sketch is generated from its original color logo by grayscale conversion, a 5×5 Gaussian blur (σ=1.1), high-pass subtraction, fivefold response scaling, and intensity inversion.

**Anime:** We also use the Anime Sketch Colorization Pair Dataset from Danbooru [28] to evaluate the applicability of our method to anime images. The dataset was released by Kim on Kaggle in 2018 and contains paired anime color images and their corresponding sketches; 300 images are selected for testing and the remaining images are used for training.

#### 4.1.2. Implementation Details

We implement the proposed model using PyTorch 2.8.0 and train it on a single NVIDIA RTX 4090 GPU (NVIDIA Corporation, Santa Clara, CA, USA). All input images are resized to 256×256, and the batch size is set to 32. The model is trained for 50 epochs. The generator, Color Style Extractor, and discriminator are optimized using Adam with an initial learning rate of $1\times 10^{-4}$ and $\beta_{1}=0.5$, $\beta_{2}=0.999$. We adopt a learning rate schedule combining linear warm-up and cosine decay. The learning rate is linearly warmed up during the first 500 iterations and then gradually decayed to $2\times 10^{-6}$ following a cosine schedule. For experiments that require model training, we conduct five independent runs with different random seeds and report the mean and standard deviation.

In our reference-based setting, the reference image primarily provides color conditions, whereas the input sketch provides structural information. During training, the original color image paired with each sketch is used both as the reference image and as the reconstruction target, supervising the model to learn reference color extraction and propagation. This training strategy is similar to existing reference-guided sketch colorization methods [29]. Specifically, the Color Style Extractor obtains global color information and spatial color features from the reference image: the former controls the overall color style through AdaIN, while the latter is used by SRSA for region-level color selection. Since AdaIN models global statistics of reference features rather than structural or pixel-wise correspondence between the reference and target, using paired references during training does not restrict inference to references with the same structure. We further evaluate this property using cross-image references in Section S2 of the Supplementary Materials.

#### 4.1.3. Evaluation Metrics

We evaluate the colorization performance using five commonly used metrics: peak signal-to-noise ratio (PSNR) [30], multi-scale structural similarity (MS-SSIM) [31], learned perceptual image patch similarity (LPIPS) [32], Fréchet Inception Distance (FID) [33], and Inception Score (IS) [34].

In addition, we propose two task-specific metrics, Color Fidelity (CF) and Boundary Integrity (BI), for evaluating reference color consistency and boundary-aware colorization quality, respectively. CF jointly measures the adherence of generated colors to the reference palette and the coverage of dominant reference colors:

(19) $$\begin{array}{cc} CF & =\frac{2AC_{\mathrm{cov}}}{A+C_{\mathrm{cov}}+\epsilon },\\ A & =\frac{1}{N}\sum_{i=1}^{N}I\;\left(\min_{m}{∥o_{i}-c_{m}∥}_{\mathrm{Lab}}\leq \tau \right),\;C_{\mathrm{cov}}=\sum_{m=1}^{M}w_{m}u_{m}. \end{array}$$

Here, $o_{i}$ denotes the Lab color of the *i*-th output pixel, *N* is the number of evaluated pixels, and $c_{m}$ and $w_{m}$ denote the *m*-th dominant reference color and its proportion, respectively. *M* is the number of dominant colors, τ is the color-matching threshold, and $u_{m}$ indicates whether the corresponding color is represented in the output. *A* measures output color adherence, while $C_{\mathrm{cov}}$ measures reference color coverage. A higher CF indicates better reference color fidelity. We set the number of dominant reference colors to M=5, the CIELab matching threshold to $\tau_{c}=15$, the minimum coverage ratio to 1%, and the near-white background threshold to 245.

BI jointly measures the effective color change and its alignment with the structural boundaries of the input sketch:

(20) $$BI=\frac{∥G_{\sigma }\left(O\right)-G_{\sigma }{\left(S\right)∥}_{1}}{∥2-G_{\sigma }\left(O\right)-G_{\sigma }{\left(S\right)∥}_{1}+\epsilon }\cdot \frac{\langle G,N\rangle }{\langle G,W\rangle +\epsilon }.$$

Here, *O* and *S* denote the generated image and input sketch, respectively. $G_{\sigma }\left(\cdot \right)$ represents Gaussian smoothing, while *G* denotes the color-gradient response, and *N* and *W* denote narrow and wide bands around sketch boundaries. At 256×256, the narrow and wide radii are 1 and 6 pixels, the smoothing scale for the change term is 0.03min(H,W), and the color-gradient field is smoothed with σ=0.5. BI is used only to assess cross-boundary color leakage and boundary clarity; it does not measure reference color accuracy or overall visual quality.

#### 4.1.4. Baselines

We compare our method with nine representative approaches under their original input conditions. To avoid mixing methods designed for different tasks, we group the baselines into three protocols: no-reference colorization, generic reference style transfer, and task-specific reference colorization. For all compared methods, we use the authors’ public implementations and retain their original network architectures, loss functions, and main training procedures. All methods are retrained on the JN-Logo-v2 training set using the same train/test split and the same computing environment. We only make necessary adaptations to data loading and input organization so that the structural input is consistently represented by the Logo sketch. Pretrained modules retain their pretrained weights, while the remaining modules are initialized and trained according to the corresponding original implementations. Comparable training budgets are used when the training settings can be unified; methods with specialized training procedures retain their original strategies. Final checkpoints are selected according to the predefined training schedules rather than test-set performance.

**No-reference methods:** pix2pix [1] retains the original U-Net generator and adversarial training procedure, taking a Logo sketch as input and the paired color Logo as the target; it does not use a reference image. CycleGAN [2] retains its original generators, discriminators, and cycle-consistency training, treating sketches and color Logos as two image domains, and likewise does not use a reference image. DDColor [3] retains the ConvNeXt-L encoder, color decoder, and original Lab colorization pipeline; the encoder uses pretrained weights and the remaining modules are trained according to the original implementation without a reference image.

**Generic reference style transfer:** StyleFormer [4] retains its original network and training objective, using the Logo sketch as the content input and an RGB Logo as the style/reference input; pretrained VGG-16 is used for feature extraction and the remaining modules are retrained on JN-Logo-v2. ${\mathrm{StyTr}}^{2}$ [5] retains the VGG encoder, Transformer, embedding module, and decoder, with the Logo sketch and RGB Logo serving as the content and style/reference inputs, respectively; the VGG encoder is pretrained and the remaining modules are retrained. TSSAT [6] retains its original feature encoder, style-matching module, and decoder, using the Logo sketch as content and an RGB Logo as style/reference; its pretrained VGG network is preserved and the remaining modules are retrained on our Logo dataset.

**Task-specific reference colorization:** Cao et al. [23] retain the sketch encoder, reference-guided generator, discriminator, and original losses, using the Logo sketch as the structural input and an RGB Logo as the reference; pretrained VGG-19 is used for perceptual feature extraction and the remaining modules are retrained. FlexIcon [24] retains the original reference encoder and generation network, using the Logo sketch as structural input and an RGB Logo as reference; only the data paths and input organization are adapted before retraining with the original configuration. AnimeDiffusion [9] retains its original conditional diffusion framework and U-Net; the paired-data loader is adapted so that the Logo sketch and RGB Logo are supplied as conditional and reference information, and the model is retrained using its original diffusion and Denoising Diffusion Implicit Models (DDIM) procedures.

### 4.2. Overall Performance

#### 4.2.1. Quantitative Comparison

To comprehensively evaluate logo colorization performance while respecting the different input conditions of existing methods, we report the JN-Logo-v2 results under the three protocols defined above. For each test image, all methods use the same structural input and data split, while reference images are provided only to methods whose original task formulation requires them. Table 1 therefore separates no-reference methods, generic reference style-transfer methods, and task-specific reference colorization methods rather than ranking all methods under a single protocol. Best and second-best values are marked only within each protocol.

For the no-reference protocol, pix2pix, CycleGAN, and DDColor infer colors without an additional reference image. Their results show that structural and semantic cues alone can produce plausible colorization, but they provide limited control over the target color scheme. For generic reference style transfer, StyleFormer, ${\mathrm{StyTr}}^{2}$, and TSSAT can use reference appearance information, but their designs primarily model global or local style statistics rather than explicit region-level color correspondence, which can lead to color confusion in compact or nested Logo regions.

Under the task-specific reference colorization protocol, Cao et al. [23], FlexIcon, AnimeDiffusion, and our method all receive both the Logo sketch and an additional RGB reference image. Within this protocol, our method obtains the best mean PSNR, MS-SSIM, LPIPS, FID, and CF. The HRP supplies explicit structural constraints for different regions, while SRSA selects suitable reference color statistics according to regional features, enabling stable reference color assignment without sacrificing the clean boundaries required by Logo images. BI is interpreted independently because it measures boundary alignment and color leakage rather than reference color correctness or overall perceptual quality.

#### 4.2.2. Qualitative Comparison

To further compare the practical colorization quality of different methods, we select several examples with different structures and color schemes from the JN-Logo-v2 test set for visual comparison. The results are shown in Figure 3. Overall, all methods can generate basic colorized results, but clear differences remain in reference color utilization, intra-region color consistency, and boundary preservation.

TSSAT can transfer part of the color and style information from the reference image, but it tends to introduce additional textures in complex contours or text regions, resulting in local blur and structural distortion. Cao et al. and FlexIcon preserve the main logo contours well in most examples, but the correspondence between some generated regions and the reference colors is not sufficiently accurate, which may lead to overall color shifts or similar colors being assigned to different regions. AnimeDiffusion produces relatively smooth colorization results, but some examples contain pale colors, local color diffusion, or background residues, which weaken the clear color blocks and boundaries required by logo images.

In comparison, our method better preserves the original contours across logos with different structures and assigns reference colors to the corresponding regions more accurately. In particular, for adjacent regions, small enclosed areas, and text structures, the generated results exhibit clearer boundaries and more consistent colors within each region, while reducing cross-region color diffusion and local color mixing. As shown in Figure 3, our method provides more stable reference color transfer while maintaining the structural integrity of the logo, producing results that better match the clear and uniform visual characteristics of logo images.

### 4.3. Model Complexity and Inference Efficiency

To evaluate the computational cost and inference efficiency of different methods, we compare the number of parameters, model size, GFLOPs, and per-image inference time on the JN-Logo-v2 dataset. All methods are evaluated with input images resized to 256×256.

As shown in Table 2, our method contains only 10.2 M parameters with a model size of 40.9 MB, both of which are the lowest among all compared methods and substantially smaller than Cao et al. [23], TSSAT, FlexIcon, and AnimeDiffusion. In terms of computational cost, our method requires 21.39 GFLOPs, second only to FlexIcon with 6.11 GFLOPs, while remaining lower than Cao et al. [23], TSSAT, and AnimeDiffusion. The per-image inference time of our method is 15.44 ms, which is close to Cao et al. [23] at 13.20 ms and FlexIcon at 15.01 ms, while being considerably faster than TSSAT and AnimeDiffusion. These results demonstrate that our method maintains low model complexity and computational cost while achieving high inference efficiency, providing a favorable balance between model size and colorization performance.

### 4.4. Ablation Study

#### 4.4.1. Component Ablation Study

To evaluate the contributions of the HRP and the SRSA module to logo colorization, we remove each component separately and further construct a variant without both components for comparison.

As shown in Table 3, removing both the HRP and SRSA leads to clear performance degradation on most metrics. In particular, the mean PSNR decreases from 24.28 dB to 22.77 dB, indicating that both structural region constraints and region-level color modeling are important for structure preservation and color recovery. When only SRSA is removed, the overall performance also decreases, with the mean CF dropping from 0.9187 to 0.9081. This shows that although the HRP still provides region information, the reference colors cannot be fully utilized without region-specific color selection. When only the HRP is removed, LPIPS and FID also degrade, demonstrating that explicit region structure is important for stable color propagation and boundary preservation.

The visual results in Figure 4 further support these observations. When both components are removed, some regions exhibit inaccurate color assignment, incomplete color blocks, or color interference between adjacent regions. Retaining the HRP while removing SRSA preserves the overall structure relatively well, but the colors of some local regions still deviate from the reference image. Retaining SRSA without the HRP allows the model to use reference color information, but color diffusion and unclear boundaries still occur in small or adjacent regions. In comparison, the complete model consistently preserves the region structure of the logo and assigns reference colors to more appropriate locations across all three examples.

#### 4.4.2. Comparison of Different Region Priors

To further analyze the influence of different region priors on logo colorization, we compare Region Prior [10], Semantic Prior [35], SAM Prior [36], and the proposed HRP as auxiliary structural information, while keeping the remaining network architecture and training settings unchanged.

As shown in Table 4, the proposed HRP achieves the best mean results on PSNR, MS-SSIM, LPIPS, CF, and BI. In particular, it reaches 24.28 dB in PSNR and 0.9187 in CF, indicating that the HRP representation can better preserve structural relationships in logos and improve the correspondence between reference colors and target regions. Semantic Prior achieves the best IS, while SAM Prior obtains the best FID.

As shown in Figure 5, Region Prior provides basic region separation, but noticeable background color diffusion can occur in some complex examples, and the color organization of local regions is less stable. Semantic Prior introduces high-level semantic information to assist color assignment, but its boundaries remain less precise for small enclosed regions and adjacent graphic elements. SAM Prior can provide relatively complete region segmentation, but its masks mainly describe region boundaries and do not explicitly encode the outer-to-inner nesting hierarchy among regions. Consequently, for Logos with multiple nested layers, some inner regions are more difficult to distinguish consistently and their reference color correspondence can become unstable. In contrast, the proposed HRP jointly encodes hierarchical relationships and spatial region structure, providing a more stable basis for distinguishing nested regions and assigning reference colors. Across all three examples, it produces more stable color assignment and better structural preservation.

### 4.5. Comparison on the Anime Dataset

To further evaluate the applicability of our method to different image types, we conduct comparative experiments on the Anime dataset. As shown in Table 5, our method achieves the best mean results on PSNR, MS-SSIM, FID, and CF. In particular, PSNR and MS-SSIM reach 18.85 dB and 0.9068, respectively, while FID decreases to 98.74 and CF reaches 0.8731. Our method also obtains the second-best mean results on LPIPS and BI. Compared with logo images, anime line drawings usually contain more complex human structures, clothing textures, and a larger number of small regions, which impose greater challenges on color correspondence and boundary preservation. These results show that our method can still preserve structural information well on complex line drawings while effectively utilizing the color features from the reference image.

The visual results in Figure 6 further illustrate the differences among different methods on complex anime line drawings. TSSAT can transfer the overall color tone of the reference image, but color mixing and local texture interference appear in some examples. Cao et al. can complete the colorization of major regions, but unstable color correspondence still occurs in detailed areas such as hair and clothing. FlexIcon produces relatively weak colorization in some examples, with several regions failing to fully utilize the color information from the reference image. AnimeDiffusion generates relatively smooth results, but the color assignments of some regions still differ from those in the reference image.

For real-world Logo sketches with moderate hand-drawn deformation, HRP can still extract corresponding region structures as long as the major contours and region connectivity remain stable, and the subsequent color assignment is therefore generally preserved. The results on the Anime dataset also indicate that the proposed method can accommodate a certain degree of structural variation. Larger deformations that substantially change the original region boundaries or topology may, however, affect region extraction and the correspondence between target regions and reference colors.

### 4.6. User Study

In addition to the objective evaluation metrics, we further conduct a user study to assess the subjective visual quality of different methods. We invite 20 participants to rate the colorization results of different methods from three aspects: Reference Color Fidelity, Region Correctness, and Overall Visual Quality. Each aspect is scored on a scale from 1 to 5, where a higher score indicates better subjective performance.

As shown in Figure 7, our method achieves the highest average score on all three evaluation aspects, indicating more stable performance in reference color preservation, region-level color accuracy, and overall visual quality. In comparison, TSSAT receives relatively lower overall scores, while Cao et al. [23], FlexIcon, and AnimeDiffusion can produce visually satisfactory results for some examples but still show variations in color correspondence and region consistency. The user study further confirms the advantage of our method in subjective visual quality.

## 5. Conclusions

This work addresses reference-based logo colorization by proposing a method that jointly exploits sketch structure and reference color information. First, we introduce an HRP that extracts region structures at different levels from the input sketch, providing explicit structural constraints for complex nested and adjacent regions. Second, we propose SRSA, which constructs a reference style library and adaptively selects suitable color statistics for different logo regions, thereby improving intra-region color consistency and reducing color interference between adjacent regions. In addition, we construct the high-quality JN-Logo-v2 dataset and introduce two task-specific metrics, CF and BI, to evaluate reference color consistency and boundary-aware colorization quality, respectively. Experimental results on JN-Logo-v2 and the Anime dataset demonstrate favorable quantitative and qualitative performance, while component ablation, prior comparison, and user studies further verify the effectiveness of the HRP and SRSA.

## Figures and Tables

**Figure 1:**
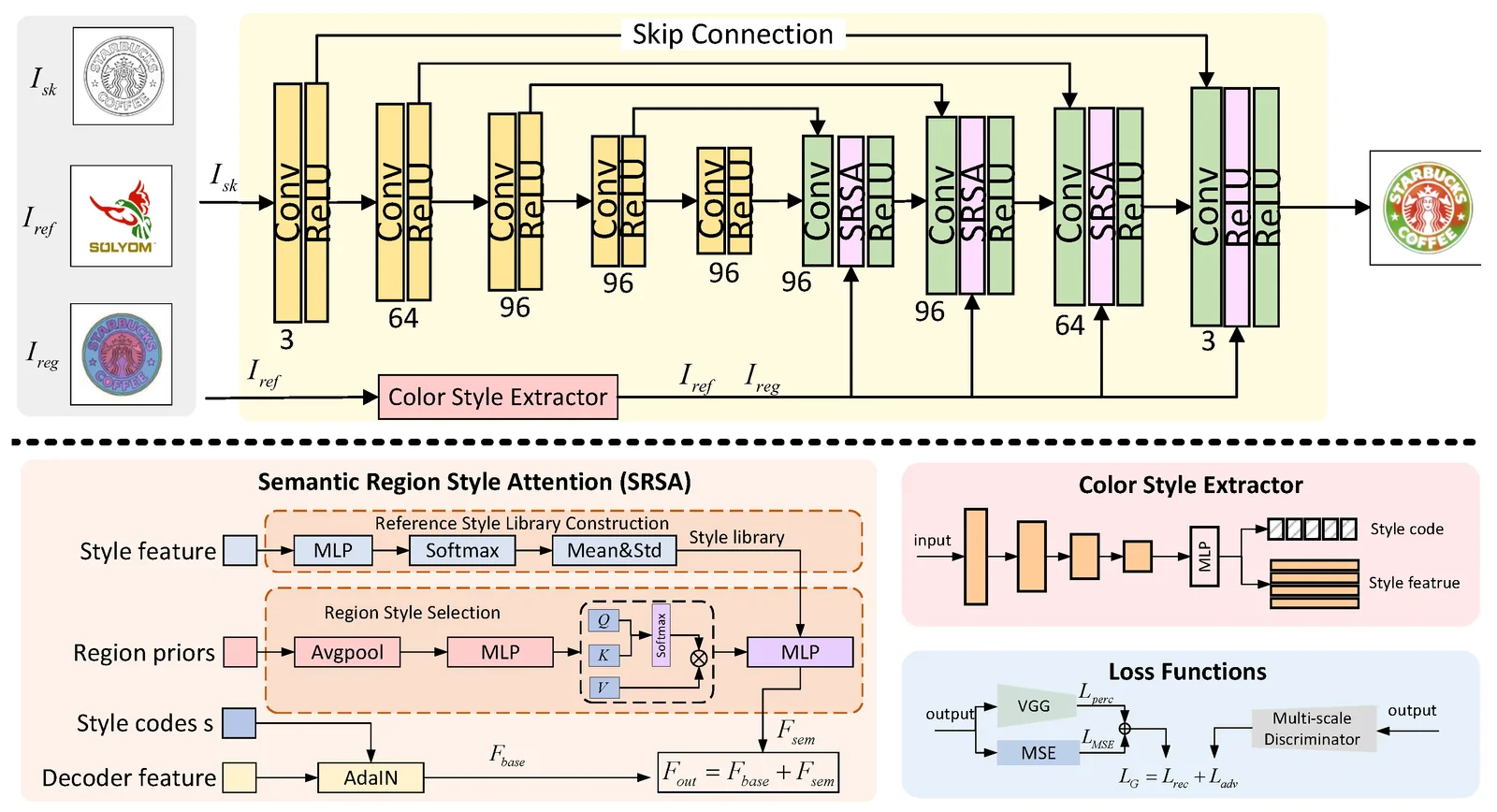
The architecture of the proposed end-to-end colorization pipeline. MLP denotes multilayer perceptron, and MSE denotes mean squared error.

**Figure 2:**
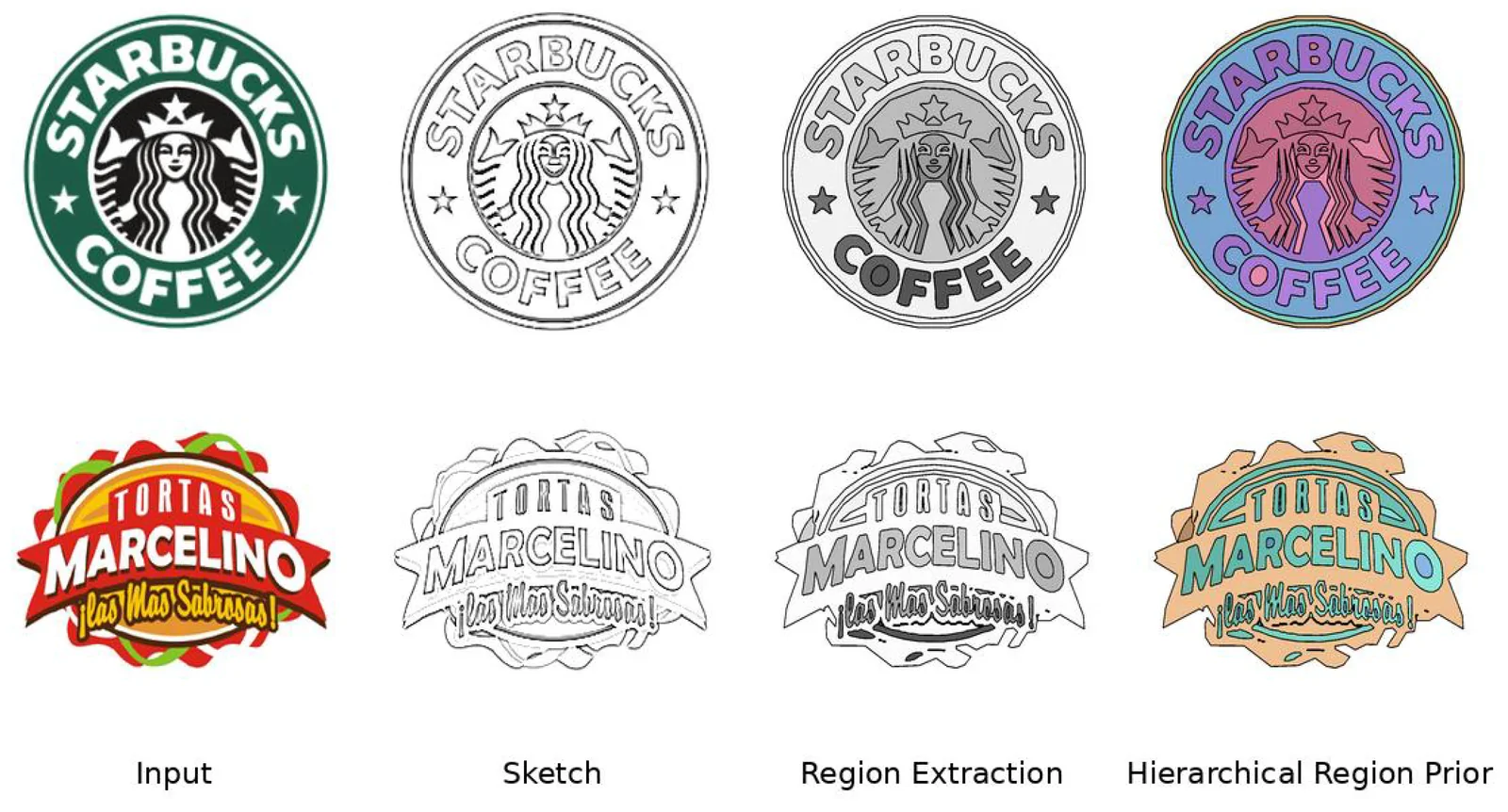
Visualization of the HRP construction process.

**Figure 3:**
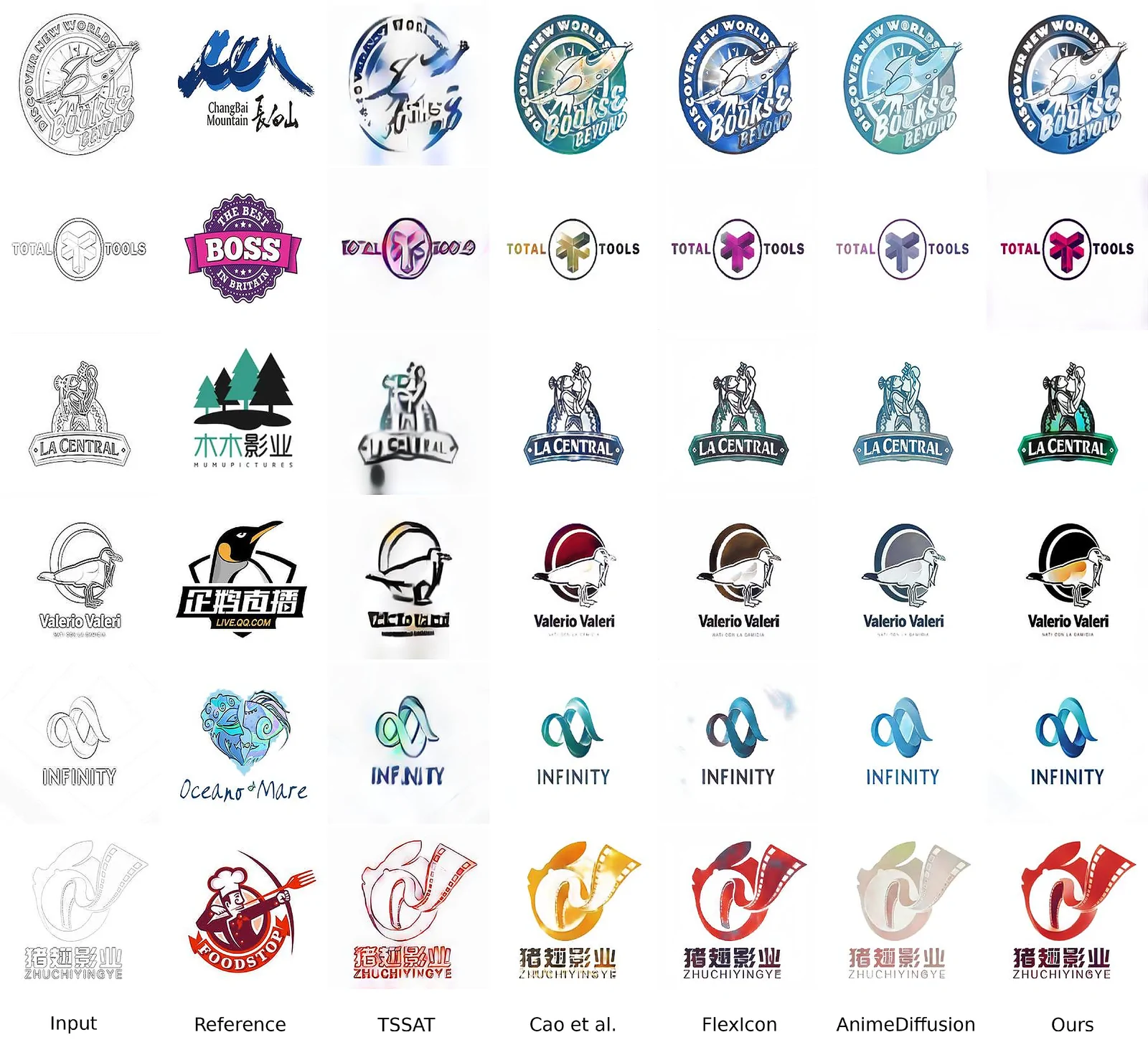
Qualitative comparison of different colorization methods on the JN-Logo-v2 dataset, including TSSAT [6], AnimeDiffusion [9], Cao et al. [23], and FlexIcon [24]. The Chinese text appearing in the original logo samples is retained as part of the source imagery. The corresponding English translations/transliterations are “Changbai Mountain” (Row 1, Reference), “Mumu Pictures” (Row 3, Reference), “Penguin Live” (Row 4, Reference), and “Zhuchi Pictures” (Row 6, Input and generated results).

**Figure 4:**
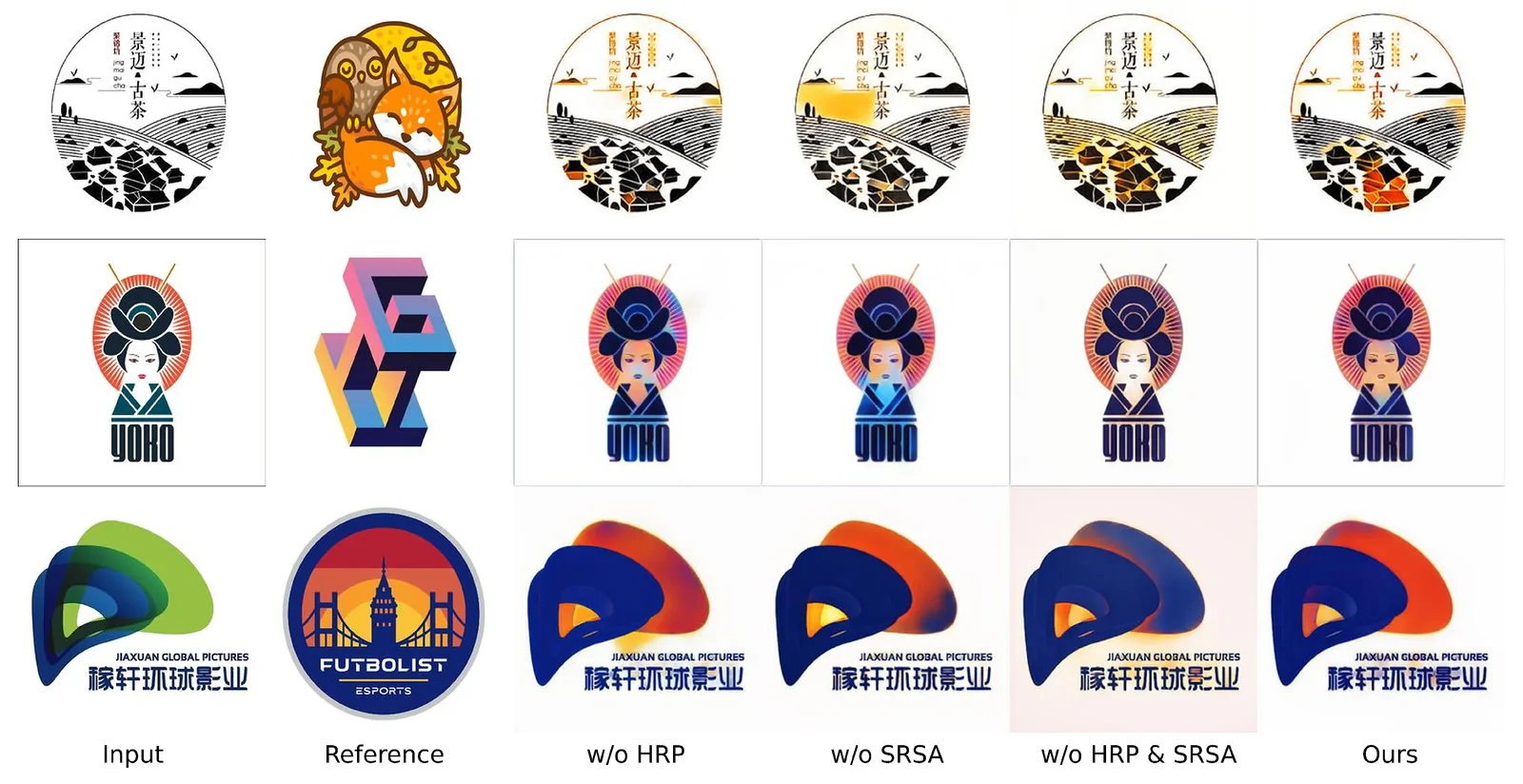
Qualitative comparison of different component ablation settings on the JN-Logo-v2 dataset. The Chinese text appearing in the original logo samples is retained as part of the source imagery. The corresponding English translations are “Jingmai Ancient Tea” (Row 1, Input and generated-result columns) and “Jiaxuan Global Pictures” (Row 3, Input and generated-result columns).

**Figure 5:**
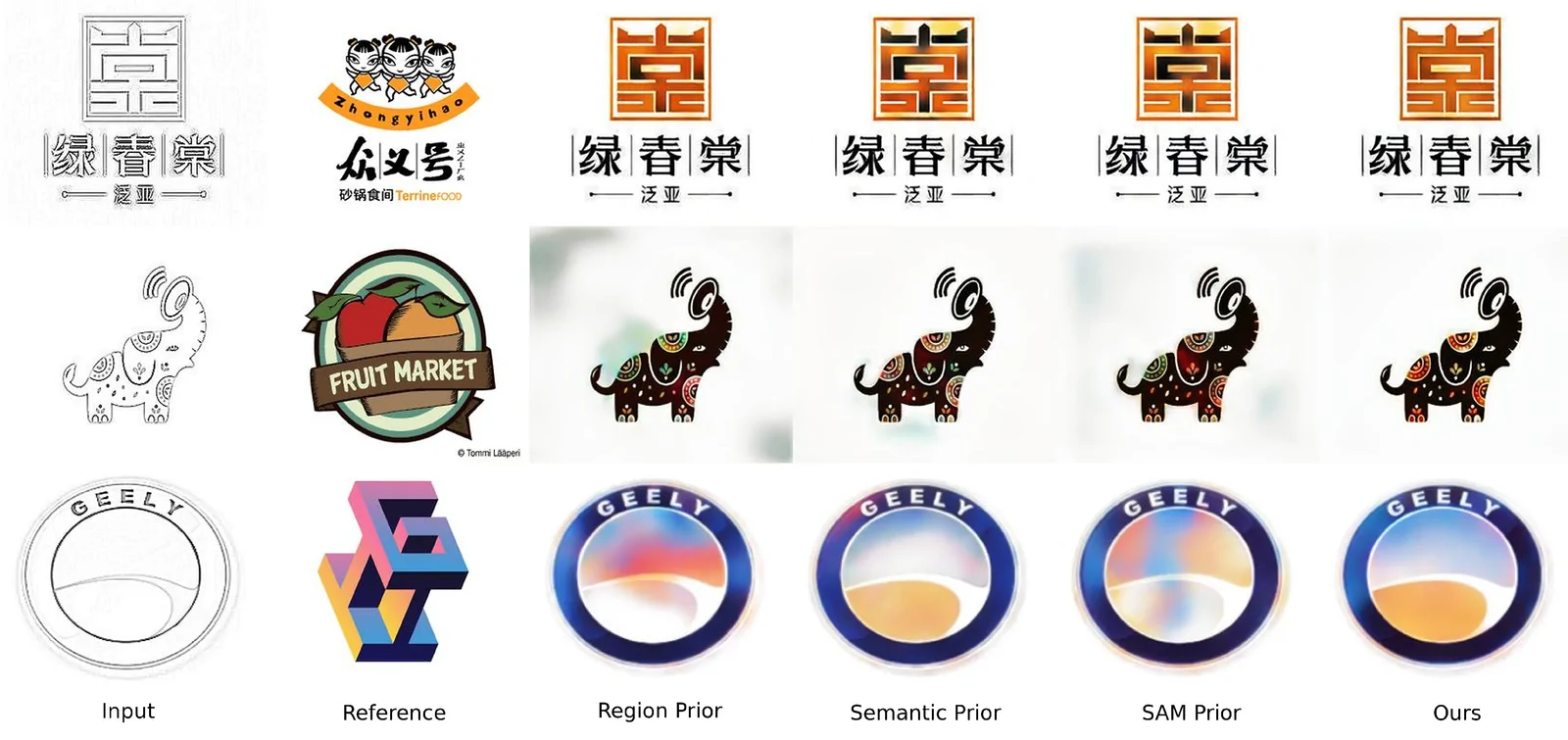
Qualitative comparison of different region priors on the JN-Logo-v2 dataset, including Region Prior [10], Semantic Prior [35], Segment Anything Model (SAM) Prior [36], and our HRP. The Chinese text appearing in the original logo samples is retained as part of the source imagery. The corresponding English translations/transliterations are “Green Spring Hall” and “Pan-Asia” (Row 1, Input and output columns), and “Zhongyihao” and “Shaguo Shijian” (Row 1, Reference).

**Figure 6:**
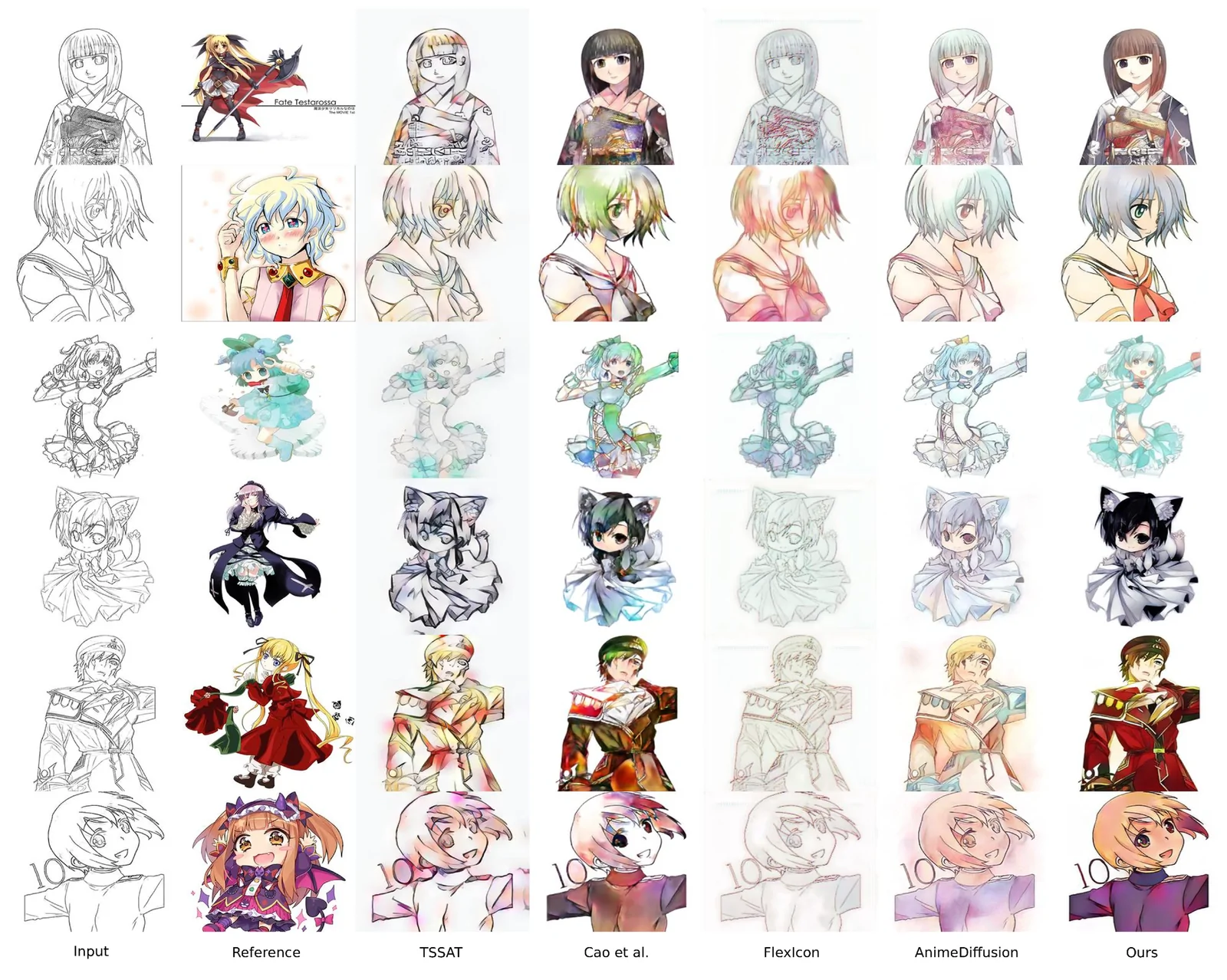
Qualitative comparison of different colorization methods on the Anime dataset, including TSSAT [6], AnimeDiffusion [9], Cao et al. [23], and FlexIcon [24].

**Figure 7:**
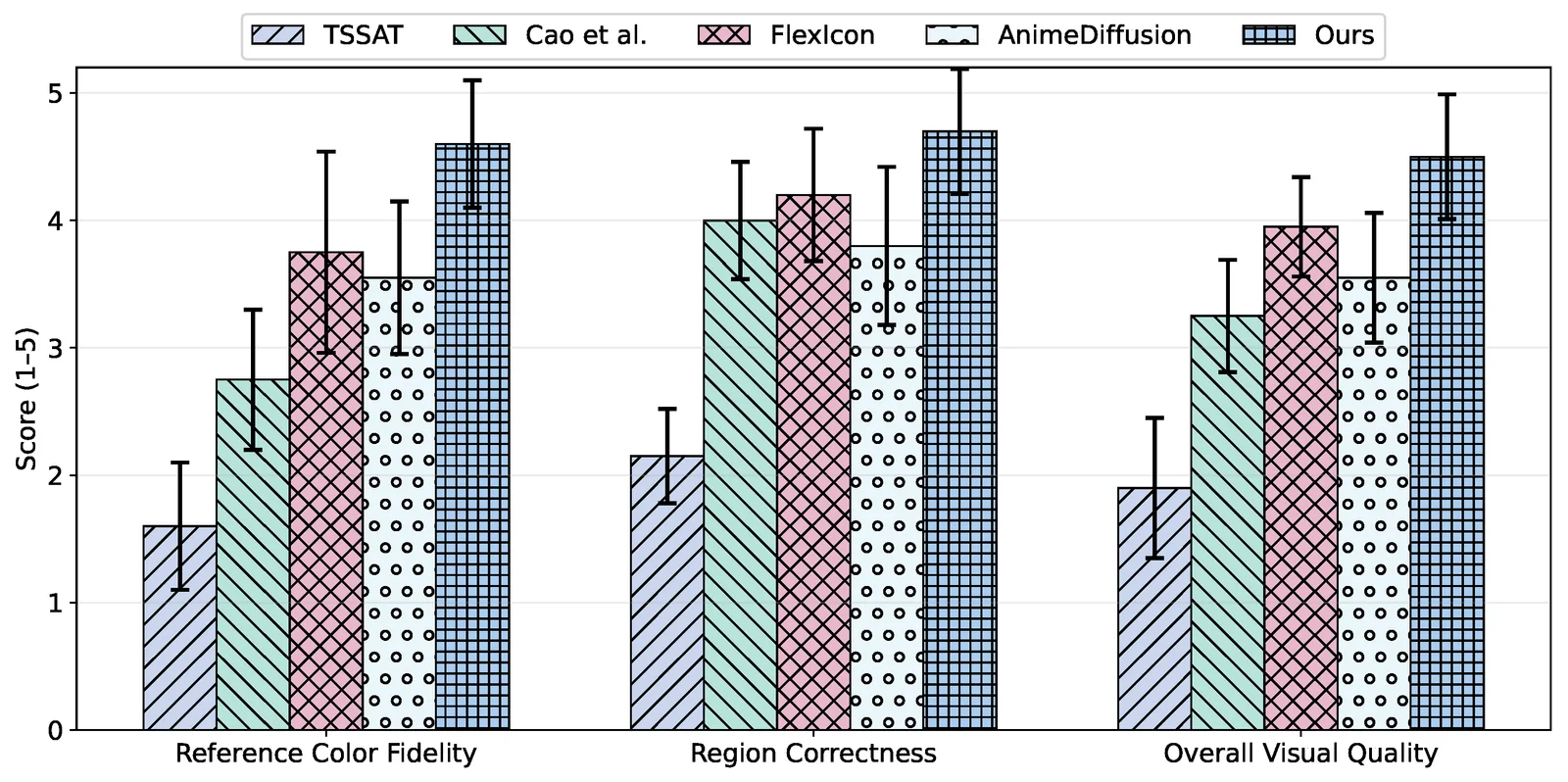
User study results of different methods, including TSSAT [6], AnimeDiffusion [9], Cao et al. [23], and FlexIcon [24], in terms of Reference Color Fidelity, Region Correctness, and Overall Visual Quality.

**Table 1:** Quantitative comparison on the JN-Logo-v2 dataset grouped by the original input condition and task protocol. “Ref.” indicates whether an additional reference image is used at inference. All results are reported as mean ± standard deviation over five independent runs. Best and second-best results are highlighted in bold and underlined only within the same protocol. ↑ indicates that a higher value is better, while ↓ indicates that a lower value is better.

Protocol	Method	Ref.	PSNR↑	MS-SSIM↑	LPIPS↓	FID↓	IS↑	CF↑	BI↑
A. No- Reference	pix2pix	×	12.24 ± 0.52	0.1939 ± 0.0537	0.6517 ± 0.0143	282.94 ± 10.91	**3.0231 ± 0.3187**	0.2252 ± 0.0597	**0.5237 ± 0.0689**
	CycleGAN	×	13.65 ± 0.71	0.5158 ± 0.0649	**0.2739 ± 0.0176**	**92.05 ± 15.48**	2.4623 ± 0.4631	0.2391 ± 0.0928	0.2507 ± 0.1387
	DDColor	×	**14.61 ± 0.31**	**0.5281 ± 0.0341**	0.2756 ± 0.0023	96.12 ± 3.67	2.5706 ± 0.1096	**0.5113 ± 0.0506**	0.0391 ± 0.0574
B. Generic Reference Style Transfer	StyleFormer	✓	13.08 ± 0.35	0.5360 ± 0.0427	0.2835 ± 0.0079	102.89 ± 5.12	2.3523 ± 0.1683	0.6945 ± 0.0709	0.2918 ± 0.0693
	${\mathrm{StyTr}}^{2}$	✓	15.88 ± 0.28	0.6555 ± 0.0389	**0.1377 ± 0.0054**	**80.15 ± 3.34**	2.8593 ± 0.1957	0.6300 ± 0.0431	0.2505 ± 0.0561
	TSSAT	✓	**17.38 ± 0.41**	**0.7973 ± 0.0413**	0.1941 ± 0.0064	109.26 ± 7.86	**3.0293 ± 0.2762**	**0.8670 ± 0.0548**	**0.3525 ± 0.0642**
C. Task-Specific Reference Colorization	Cao et al.	✓	21.95 ± 0.59	0.8887 ± 0.0496	0.0950 ± 0.0075	91.21 ± 8.93	**3.6126 ± 0.3348**	0.6384 ± 0.0663	0.3639 ± 0.1078
	FlexIcon	✓	19.73 ± 0.46	0.8361 ± 0.0518	0.0864 ± 0.0087	71.78 ± 4.79	3.2221 ± 0.2289	0.6415 ± 0.0727	0.4038 ± 0.0615
	AnimeDiffusion	✓	20.44 ± 0.94	0.8595 ± 0.1097	0.1566 ± 0.0202	110.81 ± 22.18	2.7440 ± 0.7426	0.4918 ± 0.1364	**0.5238 ± 0.1647**
	**Ours**	✓	**24.28 ± 0.23**	**0.9572 ± 0.0326**	**0.0170 ± 0.0029**	**65.54 ± 2.63**	2.8059 ± 0.1794	**0.9187 ± 0.0415**	0.3700 ± 0.0479

**Table 2:** Comparison of model complexity and inference efficiency on the JN-Logo-v2 dataset. Computational cost is reported in giga floating-point operations (GFLOPs). The reported inference time refers to the end-to-end inference time per image at a resolution of 256×256. ↓ indicates that a lower value is better. The best and second-best results are highlighted in bold and underlined, respectively.

Method	Params (M)↓	Model Size (MB)↓	GFLOPs↓	Inference Time (ms)↓
Cao et al. [23]	84.0	336.0	26.15	**13.20**
TSSAT [6]	23.5	94.1	98.37	56.90
FlexIcon [24]	76.6	306.7	**6.11**	15.01
AnimeDiffusion [9]	52.3	209.3	137.19	102.28
**Ours**	**10.2**	**40.9**	21.39	15.44

**Table 3:** Ablation study on the JN-Logo-v2 dataset. All results are reported as mean ± standard deviation over five independent runs. ↑ indicates that a higher value is better, while ↓ indicates that a lower value is better. The best and second-best results are highlighted in bold and underlined, respectively.

Method	PSNR↑	MS-SSIM↑	LPIPS↓	FID↓	IS↑	CF↑	BI↑
w/o HRP & SRSA	22.77 ± 0.38	0.9353 ± 0.0584	0.0399 ± 0.0112	86.09 ± 7.18	2.6726 ± 0.3098	0.8829 ± 0.0827	0.3208 ± 0.1005
w/o SRSA	23.14 ± 0.29	0.9489 ± 0.0457	0.0238 ± 0.0046	78.50 ± 4.02	2.7147 ± 0.1617	0.9081 ± 0.0626	0.3387 ± 0.0734
w/o HRP	23.79 ± 0.34	0.9412 ± 0.0519	0.0329 ± 0.0071	72.39 ± 5.79	2.7508 ± 0.2371	0.8982 ± 0.0751	0.3518 ± 0.0886
**Ours**	**24.28 ± 0.23**	**0.9572 ± 0.0326**	**0.0170 ± 0.0029**	**65.54 ± 2.63**	**2.8059 ± 0.1794**	**0.9187 ± 0.0415**	**0.3700 ± 0.0479**

**Table 4:** Comparison of different priors on the JN-Logo-v2 dataset. All results are reported as mean ± standard deviation over five independent runs. ↑ indicates that a higher value is better, while ↓ indicates that a lower value is better. The best and second-best results are highlighted in bold and underlined, respectively.

Method	PSNR↑	MS-SSIM↑	LPIPS↓	FID↓	IS↑	CF↑	BI↑
Region Prior	24.19 ± 0.24	0.9565 ± 0.0364	0.0174 ± 0.0038	66.22 ± 2.86	2.7796 ± 0.1458	0.9162 ± 0.0497	0.3690 ± 0.0607
Semantic Prior	23.93 ± 0.33	0.9521 ± 0.0483	0.0185 ± 0.0061	66.67 ± 4.62	**2.8168 ± 0.2197**	0.9099 ± 0.0651	0.3642 ± 0.0775
SAM Prior	24.12 ± 0.27	0.9557 ± 0.0402	0.0176 ± 0.0050	**65.34 ± 3.53**	2.7921 ± 0.1934	0.9174 ± 0.0564	0.3698 ± 0.0666
**Ours (HRP)**	**24.28 ± 0.23**	**0.9572 ± 0.0326**	**0.0170 ± 0.0029**	65.54 ± 2.63	2.8059 ± 0.1794	**0.9187 ± 0.0415**	**0.3700 ± 0.0479**

**Table 5:** Quantitative comparison on the Anime dataset. All results are reported as mean ± standard deviation over five independent runs. ↑ indicates that a higher value is better, while ↓ indicates that a lower value is better. The best and second-best results are highlighted in bold and underlined, respectively.

Category	Method	Model	PSNR↑	MS-SSIM↑	LPIPS↓	FID↓	IS↑	CF↑	BI↑
Without Reference	pix2pix	GAN	16.89 ± 0.48	0.7193 ± 0.0714	0.2005 ± 0.0123	126.14 ± 12.84	1.9468 ± 0.3928	0.5811 ± 0.0897	0.2586 ± 0.1042
	CycleGAN	GAN	14.02 ± 0.67	0.6160 ± 0.0972	0.2298 ± 0.0192	143.96 ± 17.63	2.2295 ± 0.5617	0.5508 ± 0.1215	0.2551 ± 0.1458
	DDColor	Transformer	13.79 ± 0.39	0.5414 ± 0.0453	0.2474 ± 0.0073	201.08 ± 5.07	1.6042 ± 0.2079	0.5642 ± 0.0609	0.1038 ± 0.0705
Reference- based	StyleFormer	Transformer	11.49 ± 0.44	0.5752 ± 0.0571	0.2886 ± 0.0098	178.11 ± 7.86	2.5417 ± 0.3016	0.7350 ± 0.0764	0.2627 ± 0.0880
	${\mathrm{StyTr}}^{2}$	Transformer	15.18 ± 0.31	0.6238 ± 0.0510	0.1980 ± 0.0083	134.76 ± 6.14	2.2164 ± 0.2497	0.8420 ± 0.0678	0.2976 ± 0.0793
	Cao et al.	GAN	18.05 ± 0.56	0.7709 ± 0.0754	0.1681 ± 0.0142	123.45 ± 14.09	2.4179 ± 0.4298	0.6971 ± 0.0987	**0.3372 ± 0.1154**
	TSSAT	Transformer	18.79 ± 0.42	0.8301 ± 0.0625	0.3642 ± 0.0102	124.88 ± 8.97	1.9917 ± 0.3361	0.8034 ± 0.0816	0.3161 ± 0.0935
	FlexIcon	GAN	14.53 ± 0.51	0.5548 ± 0.0673	**0.1067 ± 0.0113**	240.21 ± 10.28	1.9726 ± 0.3645	0.5754 ± 0.0851	0.2095 ± 0.0996
	AnimeDiffusion	Diffusion	16.41 ± 1.08	0.6882 ± 0.1291	0.1751 ± 0.0271	146.67 ± 26.51	**2.8175 ± 0.8127**	0.7362 ± 0.1639	0.2050 ± 0.1917
	**Ours**	GAN	**18.85 ± 0.27**	**0.9068 ± 0.0395**	0.1365 ± 0.0043	**98.74 ± 4.16**	2.3048 ± 0.2268	**0.8731 ± 0.0536**	0.3326 ± 0.0651

## Data Availability

Data are available from the corresponding author upon reasonable request.

## Supplementary Materials

The following supporting information can be downloaded at https://www.mdpi.com/article/10.3390/s26196140/s1. The accompanying Supplementary Materials contain additional experimental analyses in Sections S1–S5, Figures S1–S11, and Tables S1–S7, including SRSA reference style library size, cross-image reference colorization, controlled validation of CF and BI, HRP sensitivity and robustness, and metric sensitivity, robustness, and correlation with human judgments.
