# Supplementary material for: LogoRef: A Region-Aware Framework for Reference-Based Logo Sketch Colorization
Source: Sensors (Basel). 2026 Sep 28;26(19):6140. doi: 10.3390/s26196140 (PMC13667442; doi:10.3390/s26196140)
Supplement: Supplementary file 1 [file sensors-26-06140-s001.zip › sensors-4536897-supplementary.pdf]

Supplementary Materials: LogoRef: A Region-Aware Framework for Reference-Based Logo Sketch Colorization

Nannan Tian [1]*, Zhuolong Jiang [2]

S1. Ablation Study of P in SRSA

To analyze the influence of the reference style library size in SRSA, we conduct an additional ablation study on the hyperparameter P. In the SRSA formulation, P denotes the number of weighted groups used to construct the reference style library $\mathcal{L}$, and therefore controls how many sets of reference color statistics can be represented by SRSA. We evaluate $P \in \{2, 4, 8, 16\}$ on the JN-Logo-v2 test set, while keeping the remaining settings fixed across the ablation. The default configuration used in the main experiments is $P = 4$.

As shown in Table S1, the overall performance remains stable over a relatively wide range of P. The best mean PSNR, MS-SSIM, and BI are obtained at $P = 8$, reaching 24.218 dB, 0.9782, and 0.3856, respectively, whereas $P = 16$ achieves the best mean LPIPS, FID, IS, and CF. However, the absolute differences among the four settings are small. For example, the mean PSNR range across all settings is only 0.256 dB, while the CF and BI ranges are 0.0059 and 0.0018, respectively. These results indicate that SRSA is not highly sensitive to the exact value of P within the evaluated range.

Figure S1 further illustrates the trends of four representative metrics. Increasing P does not lead to monotonic improvements: the mean PSNR reaches its highest value at $P = 8$ and slightly decreases at $P = 16$, while mean BI also peaks at $P = 8$. Mean CF improves at $P = 16$, whereas mean LPIPS changes only marginally among the evaluated settings. This behavior suggests that the expressive capacity of the reference style library quickly approaches saturation, and a larger P mainly introduces additional style groups rather than consistently improving colorization quality. We therefore retain $P = 4$ as the default setting in the main experiments. This setting already provides competitive performance with a compact reference style library; increasing P from 4 to 16 increases the generator parameter count from 4.50 M to 6.28 M (approximately 40%) while yielding only marginal and non-monotonic improvements across the evaluated metrics.

Table S1. Ablation study of the reference style library size P in SRSA on the JN-Logo-v2 dataset. All results are reported as mean ± standard deviation over five independent runs. ↑ indicates that a higher value is better, while ↓ indicates that a lower value is better. The best and second-best results are highlighted in bold and underlined, respectively.

P	PSNR↑	MS-SSIM↑	LPIPS↓	FID↓	IS↑	CF↑	BI↑
2	24.107 ± 0.284	0.9776 ± 0.0371	0.0331 ± 0.0047	24.428 ± 3.214	<u>2.806 ± 0.184</u>	<u>0.9341 ± 0.0436</u>	<u>0.3849 ± 0.0527</u>
4	23.962 ± 0.231	0.9763 ± 0.0318	0.0335 ± 0.0039	24.193 ± 2.746	2.781 ± 0.157	0.9320 ± 0.0389	0.3843 ± 0.0462
8	**24.218 ± 0.267**	**0.9782 ± 0.0346**	<u>0.0329 ± 0.0043</u>	<u>24.166 ± 3.087</u>	2.787 ± 0.171	0.9337 ± 0.0417	**0.3856 ± 0.0498**
16	<u>24.159 ± 0.319</u>	<u>0.9778 ± 0.0403</u>	**0.0326 ± 0.0051**	**24.103 ± 3.592**	**2.829 ± 0.213**	**0.9379 ± 0.0478**	0.3838 ± 0.0571

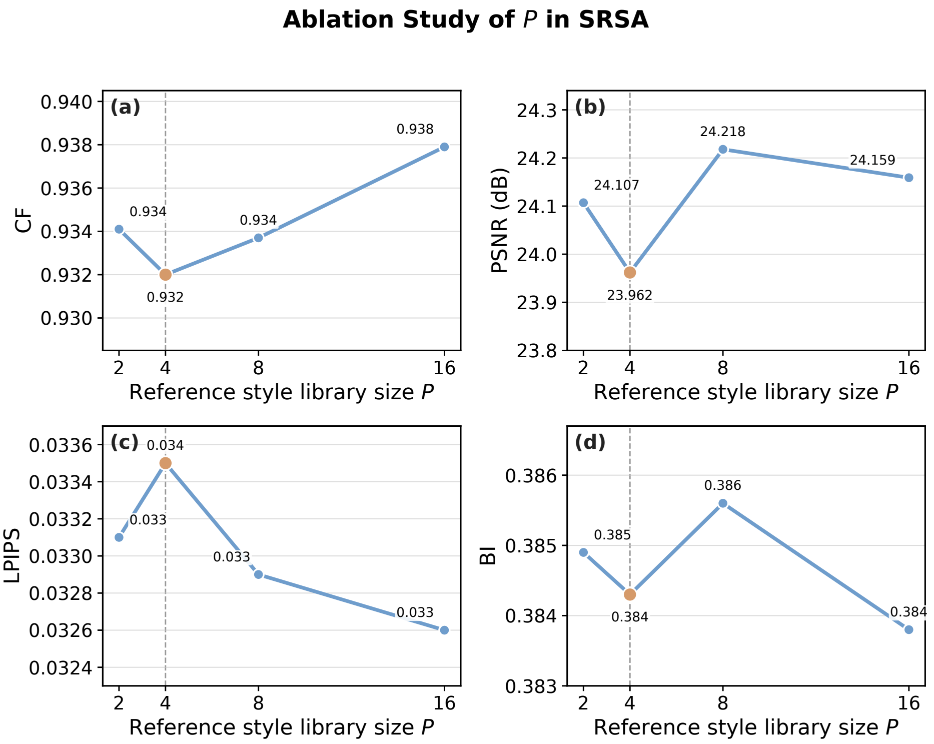

Figure S1. Influence of the reference style library size P in SRSA on CF, PSNR, LPIPS, and BI. The dashed line indicates the default setting $P = 4$ used in the main experiments.

S2. Cross-Image Reference Colorization

To further verify the color-transfer capability of the proposed method under unpaired reference conditions, we conduct a cross-image reference experiment. We select six Logo sketches and assign each of them a color reference image from a different Logo, so that the reference image is completely different from the ground-truth color image corresponding to the input sketch. All compared reference-based methods use exactly the same sketch–reference pairs for inference, without additional training or fine-tuning. The qualitative results are shown in Fig. S2.

The results show that all methods can exploit reference colors to some extent, but clear differences remain in color transfer and region consistency. TSSAT reflects part of the reference palette, although some results are pale or locally incomplete. Cao et al. and FlexIcon colorize the main regions, but the correspondence between individual regions and dominant reference colors is not always stable. AnimeDiffusion generally preserves the overall sketch structure, while the expression of the reference palette is relatively weak in several examples. In comparison, our method consistently extracts major colors from references with different structures and assigns them to distinct regions of the input sketch. References dominated by red–blue, green, or golden-yellow color schemes produce corresponding changes in the generated Logos while the original sketch contours and region structures remain well preserved. These results demonstrate that LogoRef does not require structural consistency between the reference image and the input sketch and can perform stable reference-color transfer under cross-image references.

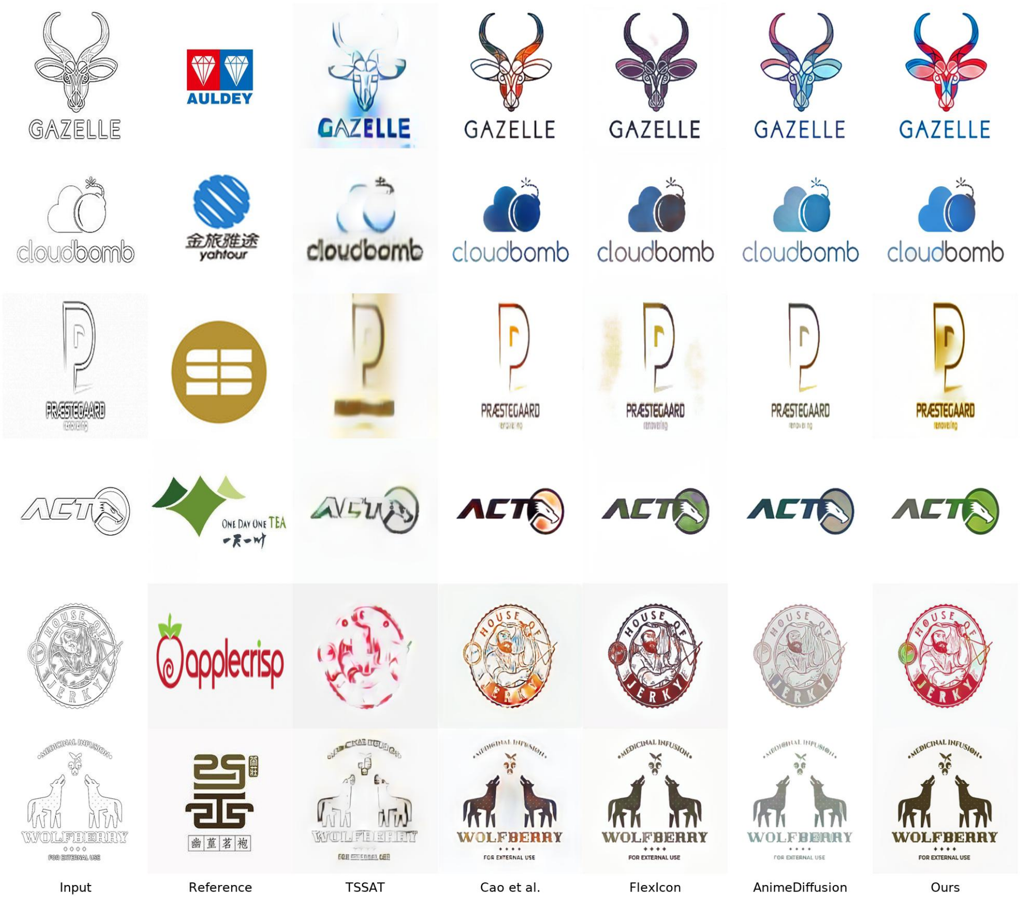

Figure S2. Qualitative comparison under the cross-image reference setting. In each row, the input sketch and the reference image come from different Logos. All reference-based methods use the same sketch–reference pair without additional training or fine-tuning.

S3. Intuitive Validation of CF and BI via Controlled Perturbations

To provide an intuitive understanding of the proposed Color Fidelity (CF) and Boundary Integrity (BI) metrics, we conduct controlled perturbation experiments on the JN-Logo-v2 test set. For each metric, we construct two perturbation families with closely matched PSNR values so that the overall reconstruction error remains comparable while the task-relevant property is deliberately changed. The quantitative results are reported as aggregate statistics, while Figures S3 and S4 provide representative visual examples.

Validation of CF. CF measures whether the generated image follows the color palette of the reference image. For the structure perturbation, the foreground is spatially translated while its color values are preserved. For the color perturbation, the spatial structure is kept fixed, whereas the dominant foreground colors are rotated in the CIELab a–b plane while preserving luminance and chroma. The perturbed colors are constrained to lie beyond the CF matching threshold ($\tau_c = 15$) from the dominant reference colors.

As shown in Table S2, the two perturbation families have nearly identical average PSNR values (19.505 versus 19.565 dB). MS-SSIM and LPIPS also respond to the perturbations, but CF exhibits a much more pronounced task-specific separation: CF decreases from 0.9436 under the structure perturbation to 0.4252 under the color perturbation, corresponding to a relative decrease of approximately 54.9%. This result shows that CF is particularly sensitive to reference-palette inconsistency when the overall pixel-level distortion is closely matched. Figure S3 illustrates representative examples of the two perturbation types.

Table S2. Aggregate controlled perturbation results for CF. The two perturbation families are matched to comparable PSNR values. ↑ indicates that a higher value is better, while ↓ indicates that a lower value is better.

Perturbation	PSNR↑	MS-SSIM↑	LPIPS↓	CF↑
Structure	19.505	0.9145	0.0430	0.9436
Color	19.565	0.8906	0.0769	0.4252

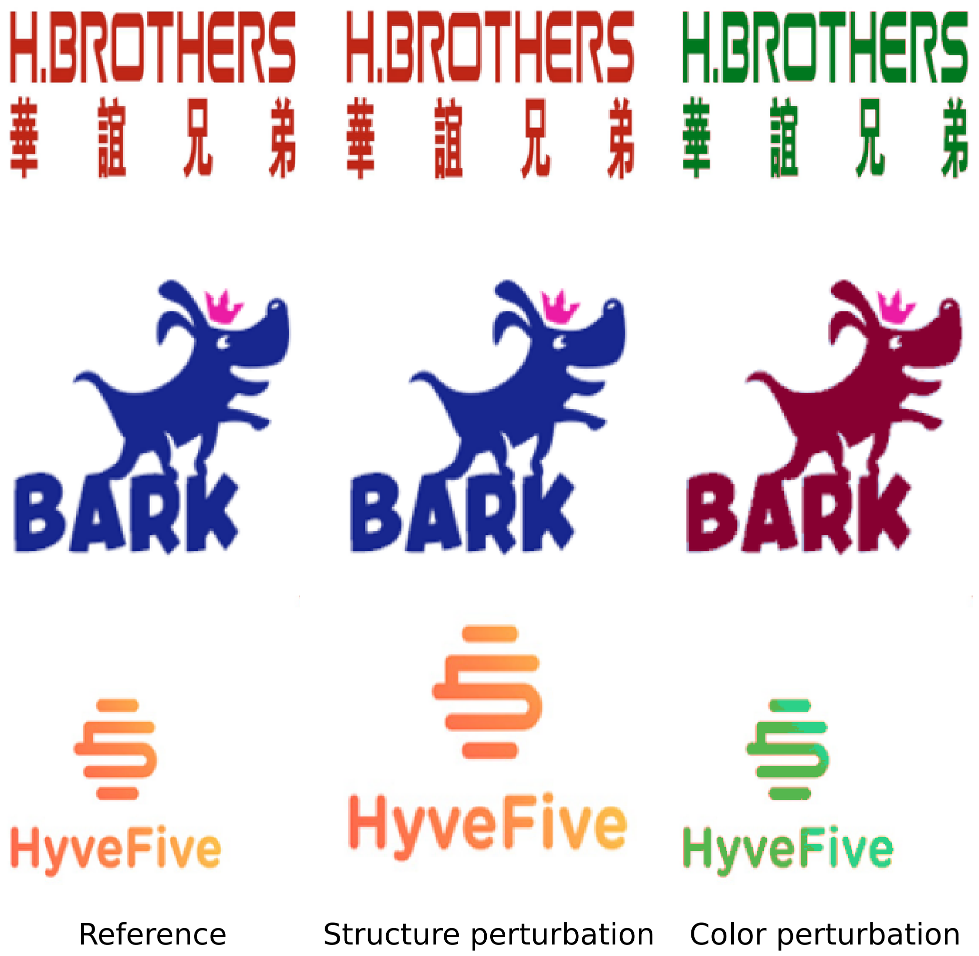

Figure S3. Representative controlled perturbation examples for CF. Structure perturbations change spatial alignment while preserving foreground colors, whereas color perturbations preserve spatial structure but alter the dominant reference colors.

Validation of BI. BI evaluates whether color transitions remain aligned with the structural boundaries of the input sketch. We construct a region-color perturbation and a boundary perturbation. The region-color perturbation changes the colors of foreground regions in the CIELab *a*–*b* plane while keeping their masks and boundaries unchanged. In contrast, the boundary perturbation translates selected region masks relative to the fixed sketch, thereby moving color transitions away from the corresponding sketch boundaries while retaining the original region colors. Perturbation pairs are retained when their PSNR difference is at most 0.3 dB and the difference in the change-related BI component is at most 0.02, reducing the influence of overall distortion magnitude and color-change amount.

Table S3 shows that the two perturbation families have closely matched average PSNR values (21.580 versus 21.558 dB). MS-SSIM decreases under the boundary perturbation, whereas LPIPS becomes lower, indicating that conventional perceptual metrics do not provide a consistent diagnosis of boundary alignment. In contrast, BI decreases from 0.4294

for the region-color perturbation to 0.3768 for the boundary perturbation, a reduction of approximately 12.3%. This result indicates that BI responds directly to color-boundary misalignment under comparable global distortion. Figure S4 provides representative visual examples.

Table S3. Aggregate controlled perturbation results for BI. Region-color and boundary perturbations are matched to comparable PSNR values. ↑ indicates that a higher value is better, while ↓ indicates that a lower value is better.

Perturbation	PSNR↑	MS-SSIM↑	LPIPS↓	BI↑
Region-color	21.580	0.8902	0.1503	0.4294
Boundary	21.558	0.8535	0.0992	0.3768

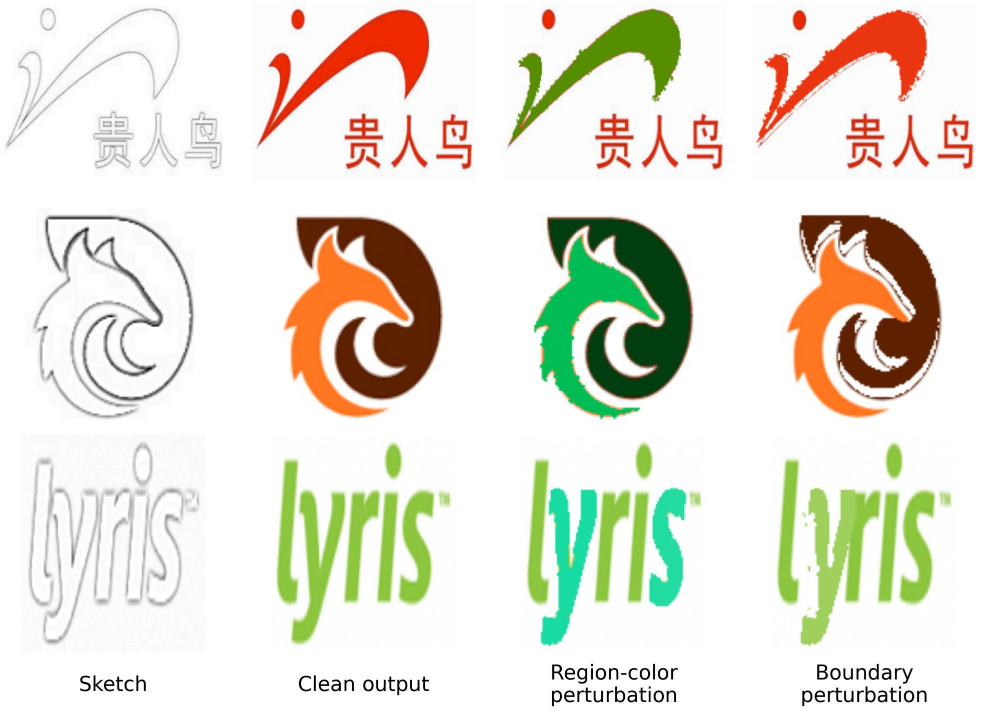

Figure S4. Representative controlled perturbation examples for BI. Region-color perturbations alter regional colors while preserving boundary alignment, whereas boundary perturbations shift selected region masks relative to the fixed sketch and introduce color-boundary misalignment.

Overall, the controlled experiments show that CF and BI provide complementary task-specific diagnostics under comparable global distortion: CF measures consistency with the reference palette, whereas BI measures the alignment between color transitions and sketch boundaries.

S4. Sensitivity and Robustness of HRP Construction

To further examine the stability of the HRP construction process, we perform additional sensitivity and robustness experiments for the major implementation choices involved in sketch preprocessing and region extraction. Unless otherwise specified, we use $K = 8$ retained regions, Otsu thresholding, one 3×3 morphological closing operation, and a minimum region area of 8 pixels. We vary one factor at a time while keeping the remaining settings unchanged. Specifically, we evaluate the maximum number of retained regions with $K \in \{2, 4, 6, 8\}$, perturb the Otsu threshold by $\pm 10\%$, remove the closing operation or enlarge the closing kernel to 5×5, and vary the minimum region area over

$\{1, 4, 8, 16, 32\}$ pixels. We additionally evaluate corrupted sketches containing contour breakage and salt noise to test the behavior of the HRP under imperfect structural inputs.

Maximum number of retained regions. Table S4 and Figure S5 show the influence of K. Increasing K improves the reconstruction-oriented metrics because more small or nested regions can be explicitly represented. PSNR increases from 21.092 dB at $K = 2$ to 23.400 dB at $K = 8$, while LPIPS decreases from 0.0397 to 0.0345 and CF increases from 0.9244 to 0.9290. BI also increases from 0.3795 to 0.3846 and becomes nearly saturated by $K = 8$. Since K directly determines the number of region masks processed by the network, we use $K = 8$ as the default setting in this sensitivity study.

Table S4. Sensitivity of the maximum number of retained HRP regions K on the JN-Logo-v2 test set. ↑ indicates that a higher value is better, while ↓ indicates that a lower value is better.

K	PSNR↑	MS-SSIM↑	LPIPS↓	FID↓	IS↑	CF↑	BI↑
2	21.092	0.9647	0.0397	29.036	2.871	0.9244	0.3795
4	22.198	0.9706	0.0367	26.604	2.801	0.9281	0.3823
6	22.910	0.9733	0.0352	25.722	2.803	0.9286	0.3839
8	23.400	0.9747	0.0345	25.018	2.790	0.9290	**0.3846**

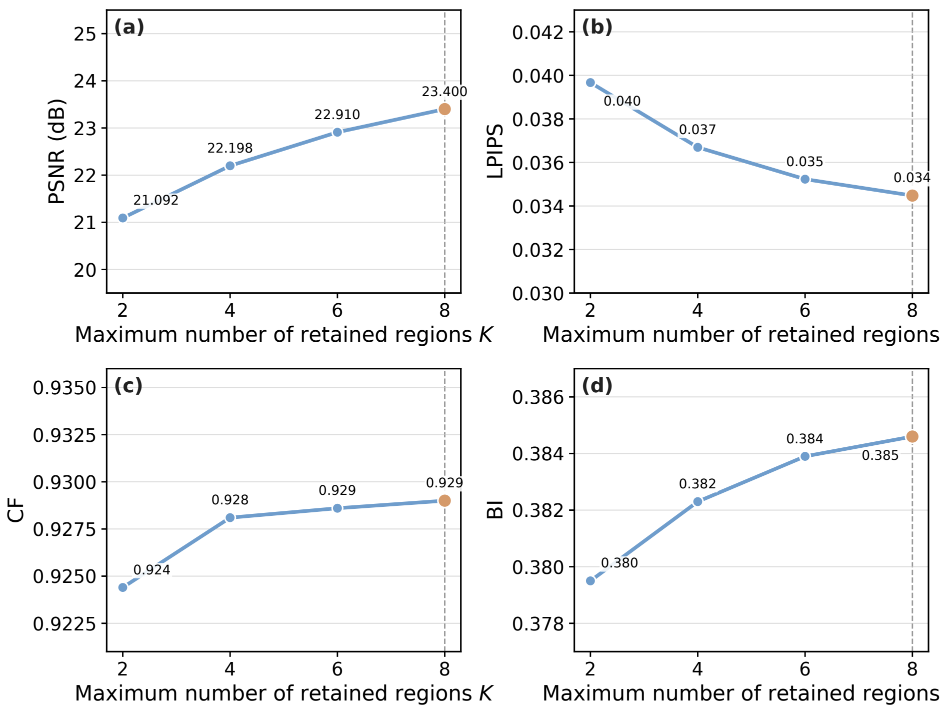

Figure S5. Influence of the maximum number of retained HRP regions K on PSNR, LPIPS, CF, and BI. Increasing the region capacity improves reconstruction quality and reference-color fidelity, while BI becomes nearly saturated at $K = 8$.

Sketch-processing parameters. The results in Table S5 indicate that HRP construction is insensitive to moderate variations of the sketch-processing parameters. Changing the threshold from Otsu−10% to Otsu+10% changes PSNR by approximately 0.01 dB relative to the Otsu setting, while CF varies by at most 0.0002 and BI by only 0.0001. Removing morphological closing or using a 5×5 closing kernel also produces only minor changes. Similarly, varying the minimum region area from 1 to 32 pixels changes PSNR by approximately 0.01 dB and leaves CF and BI effectively unchanged. These results show that the HRP is not dependent on a narrowly tuned threshold, closing kernel, or small-region filter.

Table S5. Sensitivity of HRP preprocessing and robustness to imperfect sketches. The default preprocessing uses Otsu thresholding, a 3 × 3 closing kernel, and a minimum region area of 8 pixels. The two corruption sets contain 96 contour-break and 102 salt-noise samples, respectively.

Factor	Setting	N	PSNR↑	LPIPS↓	CF↑	BI↑
Threshold	Otsu−10%	300	23.403	0.0345	0.9291	0.3845
	Otsu	300	23.400	0.0345	0.9290	0.3846
	Otsu+10%	300	23.411	0.0344	0.9292	0.3845
Closing	None	300	23.388	0.0344	0.9292	0.3844
	3 × 3	300	23.400	0.0345	0.9290	0.3846
	5 × 5	300	23.421	0.0345	0.9291	0.3846
Min. area	1 px	300	23.401	0.0345	0.9290	0.3846
	4 px	300	23.401	0.0345	0.9290	0.3846
	8 px	300	23.400	0.0345	0.9290	0.3846
	16 px	300	23.406	0.0345	0.9290	0.3846
	32 px	300	23.407	0.0345	0.9290	0.3846
Sketch quality	Clean	300	23.400	0.0345	0.9290	0.3846
	Contour break	96	20.557	0.0441	0.9247	0.3819
	Salt noise	102	20.535	0.1685	0.9305	0.3763

Robustness to imperfect sketches. Under synthetic contour breakage, CF decreases only from 0.9290 to 0.9247 and BI from 0.3846 to 0.3819, although PSNR drops to 20.557 dB. Salt noise produces a similarly stable CF value of 0.9305 and a BI of 0.3763, but LPIPS increases substantially to 0.1685. Therefore, the region-level reference-color and boundary measurements remain relatively stable under the tested corruptions, whereas severe impulse noise can still degrade overall perceptual quality. This distinction is important: the experiment supports the stability of HRP construction to moderate parameter changes and imperfect contours, but it does not imply invariance to arbitrarily severe sketch corruption. In practice, large contour gaps may merge originally separated regions and strong dense noise may create spurious components, which remains a failure mode of contour-derived HRPs.

S5. Sensitivity and Robustness of CF and BI

We further evaluate whether the proposed CF and BI metrics depend strongly on their manually selected parameters and whether they respond consistently to controlled degradations of the properties they are intended to measure. The analysis is conducted on the outputs of TSSAT, Cao et al., FlexIcon, AnimeDiffusion, and our method. For CF, the default configuration uses $M = 5$ dominant colors and a CIELab matching threshold $\tau_c = 15$. For BI, the default narrow- and wide-band radii are $r_n = 1$ and $r_w = 6$ pixels at 256×256, the change-term smoothing scale is $0.03 \min(H, W)$, and the color-gradient smoothing parameter is $\sigma = 0.5$. We vary one parameter at a time while keeping all other parameters at their default values.

Parameter sensitivity of CF. Figure S6 shows that the absolute CF value changes smoothly with both M and τ_c, as expected from their definitions. For our method, increasing M from 3 to 7 changes CF from 0.8478 to 0.9318, while increasing τ_c from 10 to 20 changes CF from 0.8029 to 0.9623. More importantly, these changes do not substantially alter the relative ordering among methods. The Spearman correlation between each non-default setting and the default ranking is between 0.90 and 1.00, indicating that the comparative conclusions drawn from CF are stable over the evaluated range rather than being determined by one narrowly selected parameter value.

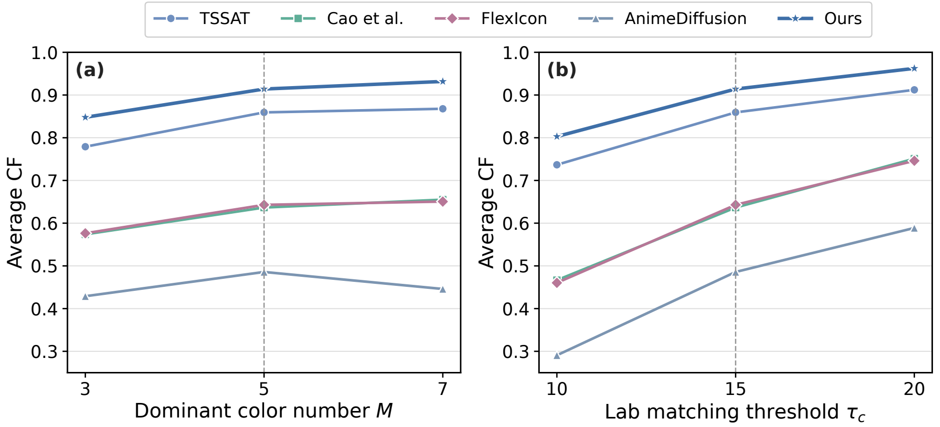

Figure S6. Parameter sensitivity of CF. We vary the number of dominant colors $M \in \{3, 5, 7\}$ and the CIELab matching threshold $\tau_c \in \{10, 15, 20\}$ using a one-factor-at-a-time protocol. The dashed vertical lines denote the default settings $M = 5$ and $\tau_c = 15$.

Parameter sensitivity of BI. Figure S7 reports the corresponding analysis for BI. The narrow-band radius has the largest influence on the absolute BI score: for our method, BI increases from 0.3682 at $r_n = 1$ to 0.4735 at $r_n = 3$. This behavior is expected because a wider narrow band tolerates larger offsets between color gradients and sketch contours. In contrast, varying the wide-band radius, change-term smoothing scale, and gradient smoothing parameter produces much smaller changes and preserves the method ranking exactly (Spearman $\rho = 1.0$). The ranking correlation for the narrow-band sweep is lower ($\rho = 0.70$), showing that r_n should be fixed and reported explicitly when BI results are compared. At 256×256, the implementation also applies a minimum effective gradient-smoothing value of 0.5; consequently, $\sigma = 0.3$ and $\sigma = 0.5$ are identical for four of the five 256×256 output sets, and this part of the sweep is interpreted conservatively.

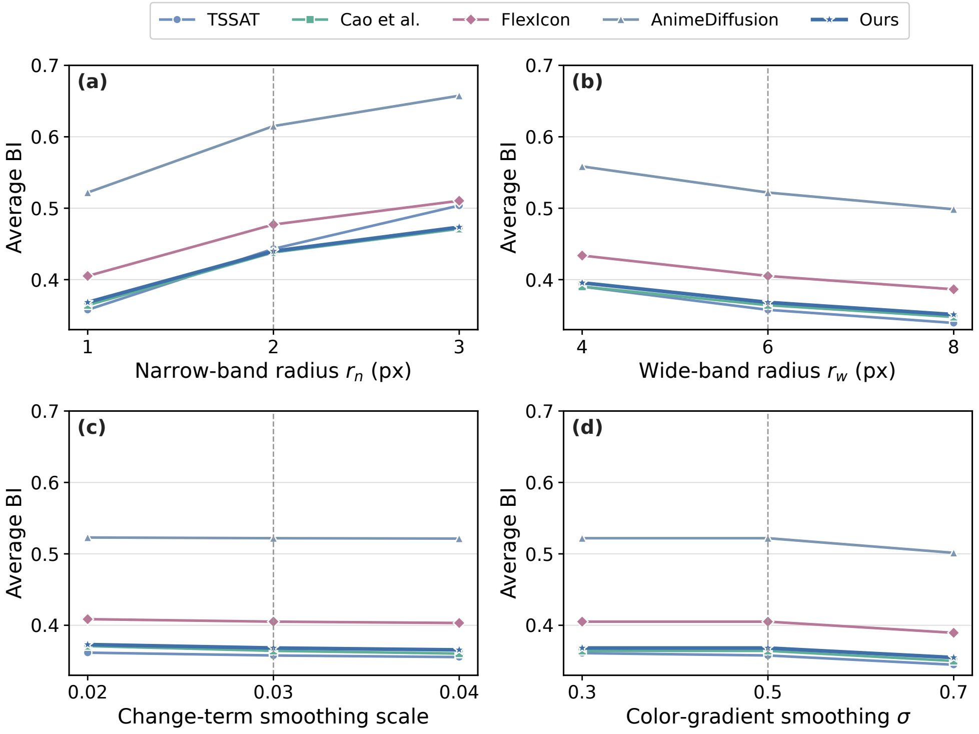

Figure S7. Parameter sensitivity of BI with respect to the narrow-band radius r_n, wide-band radius r_w, change-term smoothing scale, and color-gradient smoothing parameter σ. Dashed vertical lines indicate the default settings.

Table S6. Summary of CF and BI parameter sensitivity. The "Ours range" column gives the minimum and maximum score obtained by our method over the tested settings. Rank stability is measured by the Spearman correlation between each non-default setting and the default method ranking.

Metric	Parameter	Tested values	Ours range	Rank ρ
CF	M	3, 5, 7	0.8478–0.9318	0.90–1.00
CF	τ_c	10, 15, 20	0.8029–0.9623	0.90
BI	r_n	1, 2, 3	0.3682–0.4735	0.70
BI	r_w	4, 6, 8	0.3511–0.3956	1.00
BI	change smoothing	0.02, 0.03, 0.04	0.3656–0.3730	1.00
BI	σ	0.3, 0.5, 0.7	0.3548–0.3682	1.00

Robustness of CF. We next apply a controlled color perturbation that rotates only the foreground colors in the CIELab a–b plane by $\theta \in \{0^\circ, 15^\circ, 30^\circ, 45^\circ\}$, while preserving luminance, geometry, boundaries, resolution, and the near-white background. Ten logos are evaluated for each of the five methods, giving 200 CF evaluations. As shown in Figures S8 and S9, the CF score of our method decreases from 0.9007 ± 0.1035 at 0° to 0.5903 ± 0.3369 at 45°. The per-image Spearman correlation between perturbation angle and CF is $\rho = -0.4045$ ($p = 0.0096$). TSSAT also shows a significant negative trend ($\rho = -0.3267$, $p = 0.0396$), whereas the remaining baselines exhibit weaker and statistically non-significant trends. These results show that CF responds to controlled color deviations from the reference palette, while the response magnitude also depends on the original reference-color adherence of the evaluated outputs.

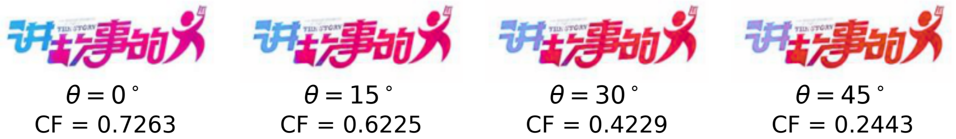

Figure S8. Example used in the CF robustness experiment. The output colors are rotated in the CIELab a–b plane while luminance and spatial structure are kept fixed. The measured CF value decreases as the hue perturbation becomes stronger.

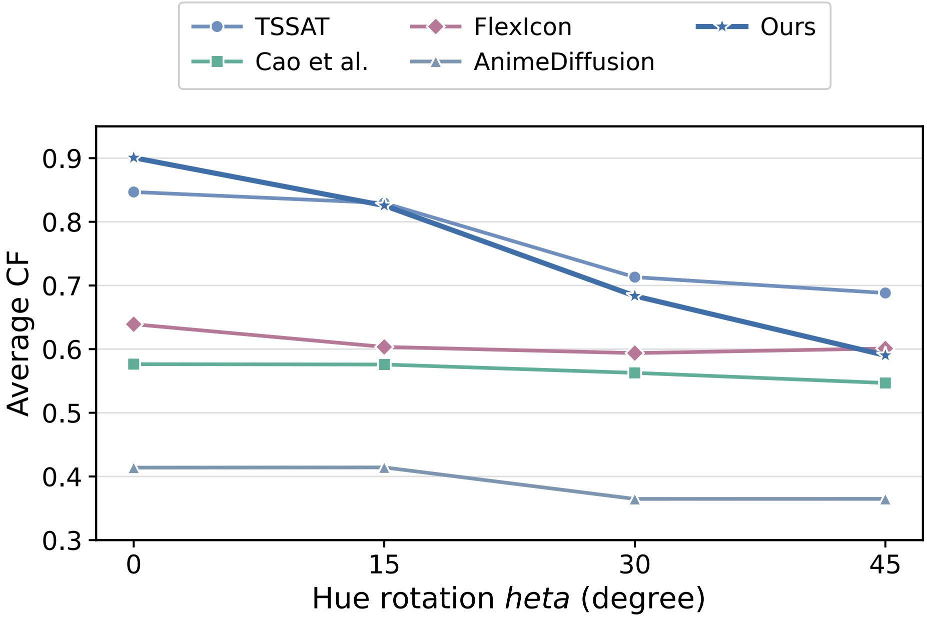

Figure S9. CF robustness under controlled hue rotation. Each curve reports the mean CF over the same ten selected logos for one method. $\theta = 0^\circ$ denotes the unperturbed output.

Robustness of BI. For BI, we perturb only the output boundaries while keeping the sketch fixed. We consider two complementary degradation axes: boundary breakage with fractions $f \in \{0, 0.1, 0.2, 0.3, 0.5\}$ and smooth zero-mean boundary jitter with amplitudes $\{0, 3, 6, 9, 12\}$ pixels at the 256×256 scale. The complete evaluation contains 300 images, five methods, and ten perturbation levels, resulting in 15,000 BI evaluations. Figure S11 shows that BI decreases monotonically with boundary breakage for all five methods, with Spearman $\rho = -1.0$ and relative decreases of 28.1–36.8% from the unperturbed output to $f = 0.5$. For our method, BI decreases from 0.3682 to 0.2430. On the jitter axis, four methods show monotonic mean decreases of approximately 13.5–15.1%; our method decreases from 0.3682 to 0.3177.

The BI decomposition further confirms that the jitter response is primarily caused by boundary misalignment rather than by a change in the amount of colored area. For our method, the boundary-alignment component B decreases from 0.6936 to 0.5969 as the jitter amplitude increases from 0 to 12 pixels, whereas the change-weight component C remains nearly constant (0.5364 to 0.5429). Thus, the decrease of BI is driven by the component that explicitly measures boundary alignment. TSSAT is the only clear exception on the jitter axis, decreasing by only 1.2%. Its released outputs contain multiple native resolutions rather than a fixed 256×256 size, while the BI band radii are scaled according to image resolution; we therefore retain these outputs without silent resizing and report this behavior explicitly rather than treating it as evidence of stronger robustness.

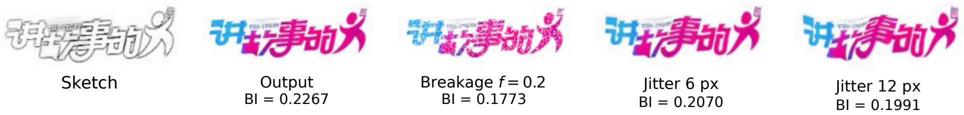

Figure S10. Example used in the BI robustness experiment. Boundary breakage removes part of the output contour band, while boundary jitter deforms the output relative to the fixed sketch. The displayed BI values decrease as the structural mismatch becomes more severe.

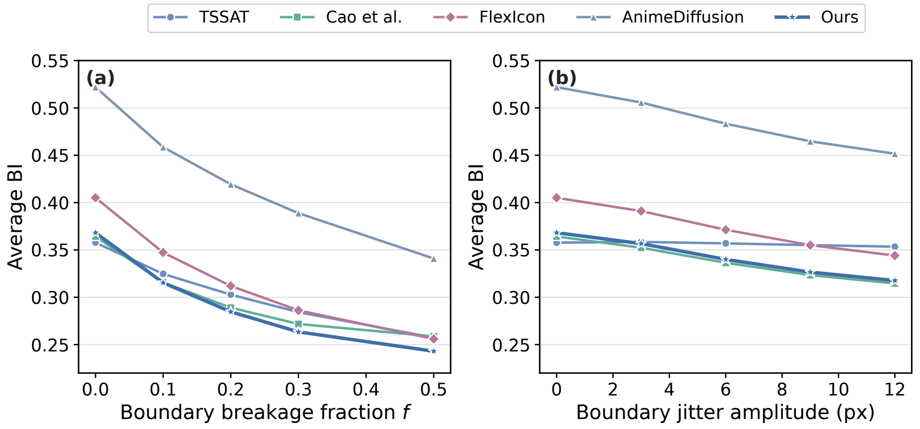

Figure S11. BI robustness under controlled boundary degradation. Left: mean BI versus boundary-breakage fraction. Right: mean BI versus boundary-jitter amplitude. The sketch remains fixed while only the output boundary is perturbed.

Correlation with human judgments. To further evaluate whether the proposed CF and BI metrics are consistent with human perception, we randomly select 50 colorization results from five representative methods and invite 20 participants to rate each sample from two aspects, Reference Color Fidelity and Boundary Integrity, on a 1–5 scale. We then compute the Pearson and Spearman correlations between the subjective scores and different objective metrics. As shown in Table S7, CF achieves the highest correlation with human judgments of reference color fidelity ($r = 0.859$, $\rho = 0.865$), while BI shows the strongest correlation with human judgments of boundary integrity ($r = 0.851$, $\rho = 0.834$). These results indicate that CF and BI are well aligned with human perception for the specific properties they are designed to measure.

Table S7. Correlation between objective metrics and human judgments. Pearson's r and Spearman's ρ are calculated over 50 colorization results. For LPIPS, a lower value indicates better quality and therefore produces negative correlations with human scores.

Objective Metric	Human Reference Color Fidelity r	Human Reference Color Fidelity ρ	Human Boundary Integrity r	Human Boundary Integrity ρ
PSNR	0.492	0.471	0.417	0.440
MS-SSIM	0.322	0.266	0.661	0.683
LPIPS	-0.370	-0.379	-0.388	-0.367
**CF**	**0.859**	**0.865**	0.321	0.295
**BI**	0.290	0.268	**0.851**	**0.834**

Overall, the sensitivity analysis shows that the comparative conclusions of CF are stable across the tested dominant-color and matching-threshold settings, while BI is stable to its wide-band and smoothing parameters but more sensitive to the narrow-band radius, which should therefore be reported explicitly. The controlled perturbation experiments further demonstrate construct-consistent responses: CF decreases when reference colors are deliberately altered, and BI decreases when output boundaries are deliberately damaged or displaced. The human-correlation analysis additionally confirms that CF and BI are strongly aligned with the corresponding human perceptual judgments. Together, these results support the use of CF and BI as interpretable task-specific metrics, while also clarifying the parameter ranges and resolution-related conditions under which they should be applied.
